# Climate‐Driven Oceanographic Changes Shape Larval Dispersal Success in the Southern Ocean

**DOI:** 10.1111/gcb.71168

**Published:** 2026-11-05

**Authors:** Fanny Ouzoulias, Alex Nalivaev, Clara Péron, Amaël Dupaix, Éric Feunteun, Félix Massiot‐Granier

**Affiliations:** ^1^ Laboratoire de Biologie des Organismes et des Écosystèmes Aquatiques (BOREA), muséum National d'Histoire Naturelle (MNHN), CNRS, IRD Sorbonne Université, Université des Antilles Paris France; ^2^ Laboratoire d'Océanographie et du Climat: Expérimentations et Approches Numériques (LOCEAN), CNRS, IRD, MNHN Sorbonne Université Paris France; ^3^ Université Libre de Bruxelles Bruxelles Belgium

**Keywords:** larval dispersal, Patagonian toothfish, polar front, recruitment, southern annular mode, variability

## Abstract

Although climate‐driven changes in ocean circulation are increasingly recognized as key drivers of marine ecosystem structure and dynamics, their consequences for the early life stages of deep‐sea fishes remain difficult to quantify. In the Southern Ocean, variability in frontal systems such as the Polar Front (PF) may influence larval dispersal and retention, with direct implications for recruitment. We investigated how interannual variability in ocean circulation and egg production shapes larval dispersal and retention in suitable recruitment areas of a large body size deep‐sea fish species, Patagonian toothfish ( *Dissostichus eleginoides* ), around the Kerguelen Plateau. We developed an integrated modeling framework which combined spatially explicit models of egg production with 24 years (2000–2023) of Lagrangian dispersal simulations driven by satellite‐derived geostrophic currents to quantify successful larval retention. Ontogenetic changes in distribution and fecundity were accounted for to model egg production, allowing retention metrics to reflect spatial heterogeneity in reproductive output. Larval retention rate in suitable recruitment areas varied from 9% to 28% over the study period. PF‐associated jet intensity was significantly associated with larval retention variability: years characterized by stronger PF‐associated circulation showed lower larval retention, with more larvae advected away from the shelf toward the eastern part of the plateau. The latter part of the time series, particularly after 2010, was characterized by stronger year‐to‐year fluctuations, a higher contribution of the North‐West recruitment area, and more frequent low‐retention years. Building on new findings on toothfish spawning dynamics and long‐term altimetry data, we showed for the first time that there is the potential for self‐recruitment in the northern part of the Kerguelen Plateau (French EEZ). We also discussed the implications for fishery management since PF‐associated indices may help interpret past recruitment trends and offer useful early‐warning for future recruitment anomalies.

## Graphical abstract

We investigate how interannual variability in ocean circulation and egg production shapes larval dispersal and retention in suitable recruitment areas of a large body size deep‐sea fish species, Patagonian toothfish ( *Dissostichus eleginoides* ) around the Kerguelen Plateau. We developed an integrated modeling framework which combined spatially explicit models of egg production with 24 years (2000–2023) of Lagrangian dispersal simulations to quantify successful larval retention. The Polar Front (PF) emerged as a key element explaining larval retention variability (c, d): years characterized by increased PF‐associated jet intensity led to less favorable conditions for larval retention, with more larvae advected away from the shelf toward the eastern part of the plateau (a), compared to years with lower PF‐associated circulation and a stronger northern pathway (b). Indices derived from Polar Front circulation metrics offer useful early warning for future recruitment anomalies.

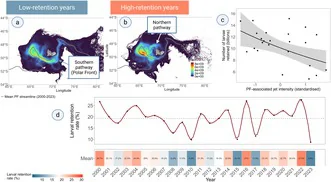

## 1 Introduction

Climate change is transforming the oceanographic conditions that govern the survival and dispersal of early life stages in many marine species. These conditions influence fundamental biological processes such as survival, growth, and reproduction across trophic levels (Hollowed et al. 2013; Pinsky et al. 2020). Survival and growth during the early life stages are especially critical as they largely determine recruitment success and population renewal (Houde 2008). Understanding how variations in oceanographic conditions influence these early stages is therefore essential for anticipating population responses to climate‐driven changes.

The Southern Ocean is experiencing pronounced changes in its circulation, with intensified westerly winds, shifts in oceanic frontal systems, and enhanced mesoscale variability (flow structures of 10–100 km and timescales of weeks to months) (Meredith and Hogg 2006; Swart and Fyfe 2012; Martínez‐Moreno et al. 2021). These changes are already altering the structure and dynamics of marine ecosystems, particularly through the influence of the Antarctic Circumpolar Current (ACC), the world's strongest ocean current, and its associated frontal systems (Constable et al. 2014). A central feature of the Southern Ocean circulation is the Polar Front (PF) (Figure 1), a major hydrographic boundary that separates Subantarctic Surface Waters from Antarctic Surface Waters (Dong et al. 2006; Pollard et al. 2002; Park et al. 2014). The PF can be identified either from the properties of the water masses it separates or from the surface circulation associated with it: traditionally, the PF is defined from hydrographic observations as the northern limit of Antarctic Winter Waters, which are characterized by a subsurface temperature minimum (Chapman et al. 2020). The PF can also be described by its associated surface circulation since ACC fronts are associated with very intense surface currents known as jets (Park et al. 2019; Chapman et al. 2020). These two definitions describe related aspects of the same frontal system, but their positions do not necessarily coincide in space and time (Chapman et al. 2020). Interacting with topographic features such as the Kerguelen Plateau (Southern Indian Ocean), the PF shapes ecological connectivity by structuring the distribution and transport of water masses and planktonic organisms (Blain et al. 2007). Around the Kerguelen Island, the PF exhibits strong seasonal and inter‐annual variability, particularly west of the Island where bathymetric constraints are weak compared to the south and east (Pauthenet et al. 2018). These dynamics influence not only physical transport but also biological processes. Several studies have shown that PF dynamics directly affect foraging behavior, demographic traits, and reproductive success in upper‐trophic predators such as penguins (Péron et al. 2012; Bost et al. 2015). The pronounced thermal gradients associated with the PF are also thought to act as a biological barrier, constraining the dispersal and spatial distribution of a wide range of marine taxa (Fraser et al. 2012). However, the consequences of PF variability for the early life stages of marine species remain difficult to quantify. This gap is particularly critical for exploited fish species, as recruitment in marine fish is strongly shaped by environmental conditions encountered during the dispersal and settlement phases of early life stages (Paulik 1973; Rothschild and Fogarty 1989).

**FIGURE 1:**
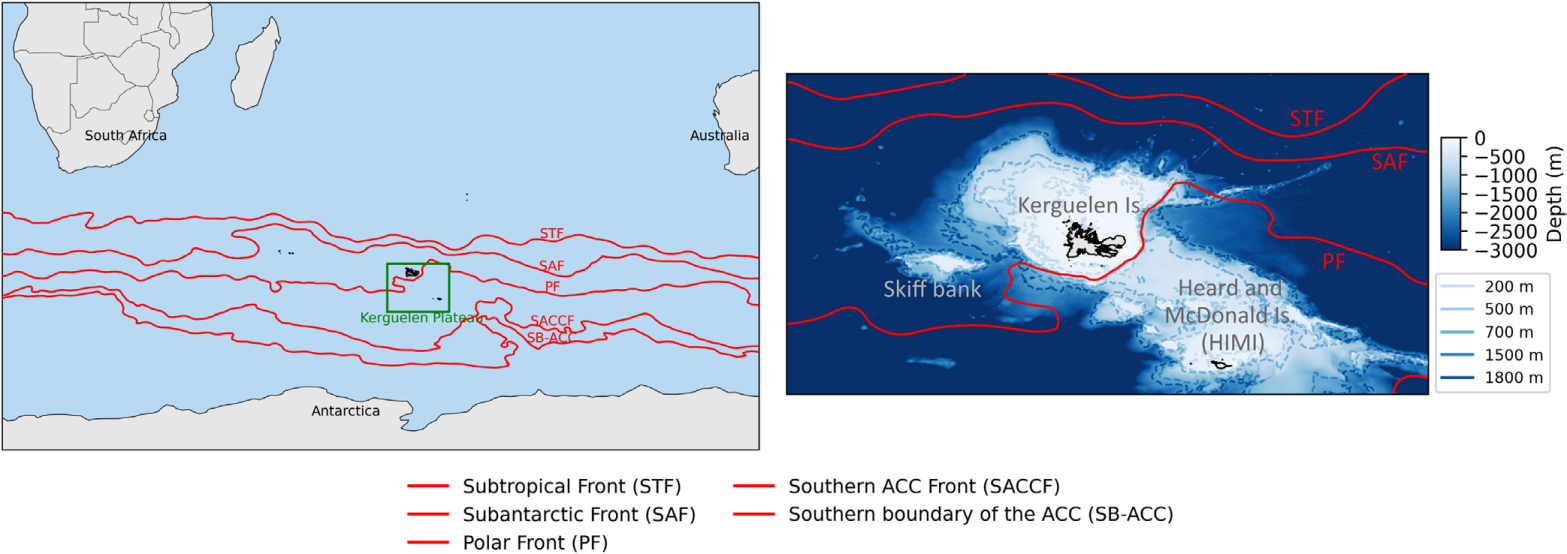
Main fronts in the Southern Indian Ocean and around the Kerguelen Plateau. Data from Park and Durand (2019) (doi: https://doi.org/10.17882/59800).

Recruitment is commonly defined as the transition from fertilized eggs to settled juveniles and is governed by several processes operating across different spatial and temporal scales (Houde 2008). One key component of recruitment is larval retention: the proportion of larvae that reach suitable recruitment areas after being dispersed by ocean currents (Cowen 2002). Biophysical models are widely used to investigate larval dispersal and connectivity, typically combining hydrodynamic simulations with Lagrangian particle tracking (Lett et al. 2010; McGeady et al. 2022). Although some approaches incorporate species‐specific traits such as growth and mortality through individual‐based modeling, a critical limitation is that most studies assume uniform spawning effort, ignoring the spatial distribution of egg production. Yet, in species where both fecundity and spatial distribution vary with body size, spatial patterns of reproductive output may significantly influence larval retention. Both ocean circulation and the spatial distribution of spawning thus play key roles in determining dispersal outcomes.

In this study, we investigated the effects of PF variability on the early life stages of a large body size and commercially exploited fish species, Patagonian toothfish ( *Dissostichus eleginoides* ). Ontogenetic changes in distribution and fecundity were accounted for in an integrated framework combining spatially explicit models of spawning effort (i.e., eggs initially released in the spawning areas) and larval dispersal models. Patagonian toothfish is a commercially valuable demersal species widely distributed across the Southern Ocean and exhibits a pronounced ontogenetic habitat shift: pelagic larvae occupy surface waters, juveniles settle on the shelf (< 200 m), and adults progressively move to depths exceeding 2000 m as they grow (Duhamel et al. 2005; Collins et al. 2010; Péron et al. 2016). Fecundity scales with body size, suggesting that the spatial distribution of large spawners could strongly affect larval retention. Despite its ecological and economic significance, the early‐life ecology of this species and its fecundity remain poorly understood. In the Falkland Islands, otolith microstructure and trace element analyses have revealed key developmental benchmarks, such as hatch timing, pelagic larval duration (PLD), and juvenile settlement timing on the shelf (Lee et al. 2024). A study by Mori et al. (2016), conducted at the scale of the Kerguelen Plateau, used satellite‐based simulations and showed that larval retention was more likely for spawners located in the northern areas (latitude < 54° S) and that spawners located in the west contributed the most to successful larval retention. The authors also hypothesized that the latitudes 51°–53° S on the western slopes of the plateau were a particular hotspot for spawning. A recent study investigated for the first time the spawning dynamics of Patagonian toothfish in the northern part of the plateau (French Kerguelen Exclusive Economic Zone (EEZ)) and found that spawning occurred mostly in June and July in the western part of Kerguelen plateau, between 48° S and 50° S at depths ranging from 1500 to 1800 m or very locally in shallower waters (500 m) (Ouzoulias et al. 2025). Further south, in the Australian EEZ around Heard and McDonald Islands (HIMI), spawning is also known to take place along the deeper western slopes of the plateau, with a depth preference between 1700 and 1800 m (Welsford et al. 2012; Yates et al. 2018). After spawning, eggs rise to the upper water column, between 0 and 200 m, where they hatch after 30–90 days (Lee et al. 2024). Larvae subsequently undergo a 3‐month dispersal period, during which they are advected by ocean currents, before settling (Koubbi et al. 1990; Evseenko et al. 1995; North 2002).

Larval retention rate is defined as the proportion of larvae that successfully settle in a suitable recruitment area by the end of the dispersal period (PLD), relative to the total number of eggs initially released in the spawning areas (i.e., spawning effort). Defining suitable recruitment areas is thus a key step when investigating the larval retention process. Several scientific bottom trawl surveys were conducted in the Kerguelen Plateau (Coghlan et al. 2025; Péron et al. 2025) and found that juvenile fish (< 2 yo) inhabited shallow waters < 150 m where they feed on crustaceans and may be more sheltered from predation than in deeper waters. Juveniles of 1 yo have been found in Kerguelen Is. fjords (Golfe des Baleiniers, around 70° E/49° S), and around Skiff bank (unpublished data).

Here, we aim to build from new findings on Patagonian toothfish spawning and long‐term altimetry data (2000–2023) to investigate how spatial and temporal variability in both spawning dynamics and ocean circulation jointly influence larval dispersal and retention of Patagonian toothfish on the Kerguelen Plateau.

Specifically, we address the following questions: (1) How does climate‐driven variability in the PF modulate inter‐annual larval retention and the relative contributions of different spawning and recruitment areas? (2) What are the dominant dispersal pathways of eggs and larvae? (3) What role do ontogenetic changes in female spatial distribution and fecundity, together with spawning phenology, play in larval retention?

We developed a new integrated framework combining spawner distribution, fecundity, and ocean circulation (Figure 2) to provide new insights on the effects of climate change on larval retention in the Southern Ocean.

**FIGURE 2:**
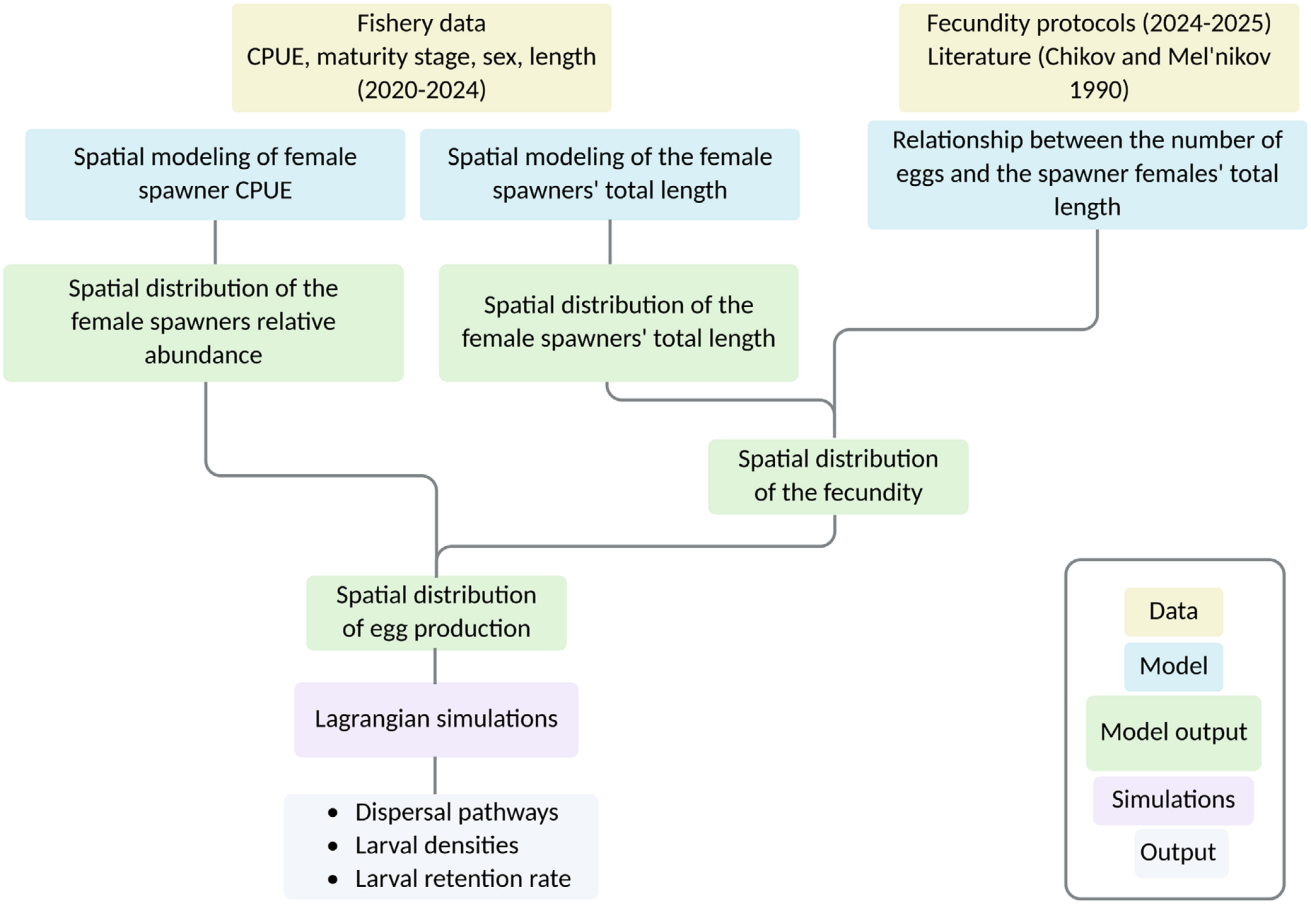
Flowchart of the methods used to estimate the total production of eggs in the spawning areas and to simulate particle trajectories. The equations of the different models are in Appendices S1.1–S1.4. CPUE: Capture Per Unit of Effort.

## 2 Materials and Methods

This study combines two integrated approaches: (1) estimating the spatial distribution of released eggs during spawning as a proxy for reproductive output; and (2) simulating particle trajectories, with each particle weighted by the corresponding number of eggs released (Figure 2).

### 2.1 Modeling Spatial Distribution of Reproductive Outputs (Egg Production)

#### 2.1.1 Fishery Data Collection

Since 2000, the Patagonian toothfish fishery in the Kerguelen EEZ has been conducted by up to nine 60‐m longliners using demersal integrated weight autolines. Gear configuration has remained consistent across vessels and seasons. For each haul, total catch and detailed metadata (position, depth, and effort) were recorded, and biological sampling was conducted by onboard observers on one or two of the five lines hauled each day. For these lines, the inspector samples fish randomly from the catch (mean = 65 individuals/line). Fish were measured for total length, sexed, and staged for maturity using macroscopic criteria (Kock and Kellermann 1991; Gasco 2010). These data were extracted from the “Pecheker” database (Martin et al. 2021), hosted and administered at the Museum national d'Histoire naturelle (MNHN).

#### 2.1.2 Spatial Distribution of the Relative Abundance of Female Spawners

To predict the abundance of female spawners, we focused on stage‐4 (ripe) females sampled during the spawning period (weeks 22–30, June–July) between 2020 and 2024. This spawning window was defined based on the phenology of ripe females quantified from fishery observer data (2020–2024), which shows a clear winter peak with the highest predicted female spawner CPUE from early June to late July (Ouzoulias et al. 2025). For each sampled line, the total number of spawners was estimated by scaling observed maturity proportions to total catch declared in numbers by the skipper.

Spatial variation in spawner abundance was modeled using a generalized linear model with spatial Gaussian Markov Random Fields (GMRFs), implemented in the sdmTMB framework (version 1.0.0.9007) (Anderson et al. 2024) available as a library in the R software (version 4.3.3). Spawner abundance was modeled assuming a Tweedie family distribution with a log link function. Models accounted for depth, slope, fishing effort (in number of hooks hauled), and spatial autocorrelation (Appendix S1.1).

Prediction uncertainty was propagated using simulation‐based draws generated with sdmTMB's predict(), which samples from the joint precision matrix of the estimated fixed and spatial random effects. Throughout, we refer to these draws as “posterior simulations.”

#### 2.1.3 Spatial Distribution of Female Spawners Average Length and Their Associated Fecundity

The same methodology as in Section 2.1.2 was used. Additional details are provided in Appendix S1.2.

We estimated fecundity based on 178 observations collected from two complementary data sources. We used published data from Chikov and Mel'nikov (1990), who measured egg diameters in 55 mature females during a bottom‐trawl expedition on the Kerguelen Plateau in March–April 1987. Data were digitized using the software *PlotDigitizer*. The second dataset was obtained during a dedicated protocol conducted on board a French commercial longliner (“*Ile de La Réunion 2*”) in 2024 (July 19 to August 31, *n* = 27 females) and 2025 (June 24 to July 11, *n* = 96 females), during which gonad volume and egg diameter were measured, enabling the calculation of the number of eggs in the gonads.

An exponential relationship between total length and fecundity was fitted using non‐linear least squares (Appendix S1.3). Uncertainty in the fitted relationship itself was quantified for visualization using a first‐order Taylor (delta‐method) approximation, but spatial uncertainty relied exclusively on posterior simulations of predicted total length.

To generate spatial estimates of fecundity, the fitted length–fecundity relationship was applied to posterior simulations of predicted total length (Appendix S1.3).

#### 2.1.4 Spatial Distribution of Reproductive Outputs (Egg Production)

To map egg production during the 2020–2024 spawning period, we combined two spatial components predicted on a 10 × 10 km grid: (i) female spawner relative abundance and (ii) female fecundity derived from the spatial total‐length model and the fitted length–fecundity relationship (Figure 2).

Uncertainty was propagated by pairing posterior simulations from both components at each grid cell. Specifically, 1000 posterior predictive simulations of female abundance were multiplied by the corresponding 1000 fecundity simulations derived from total‐length predictions (Appendix S1.4). This yielded 1000 realizations of egg production per grid cell, which were summarized by their mean and standard deviation to provide spatially explicit estimates of reproductive output and associated uncertainty.

### 2.2 Definition of the Eggs Release Area

Based on the map of egg production and on the spawning areas identified by Ouzoulias et al. (2025), we defined a spawning ground encompassing the main spawning hotspots, primarily located in the western spawning zone and around Skiff Bank, between the 1500 m and 1800 m isobaths (Figure 3).

**FIGURE 3:**
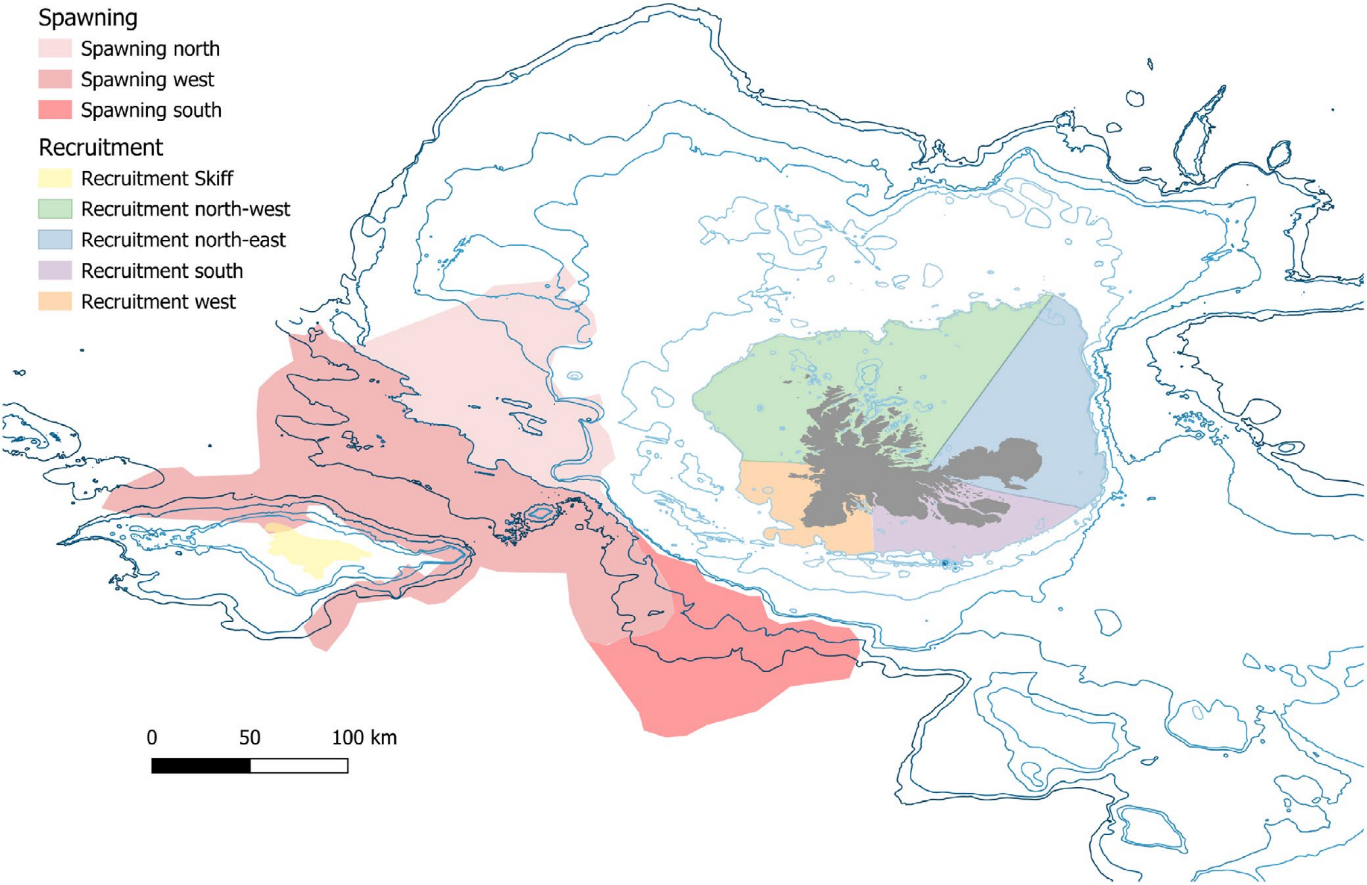
Subdivision of spawning and recruitment areas, with the 150, 500, 700, 1500, and 1800 m isobaths. Lighter shades represent shallower depths.

### 2.3 Larval Dispersal Simulations

Patagonian toothfish spawn at approximately 1500 m depth (Ouzoulias et al. 2025). Direct measurements of egg buoyancy are not available for Patagonian toothfish; we therefore used information from Antarctic toothfish ( *Dissostichus mawsoni* ). The two species show close phylogenetic kinship (Ghigliotti et al. 2007) and produce large eggs of broadly comparable size: *D. eleginoides* eggs have been reported to measure 4.3–4.7 mm in diameter, with a mean of approximately 4.5 mm (Koubbi et al. 1990; Evseenko et al. 1995), whereas *D. mawsoni* eggs measured by Parker et al. (2021) had a mean diameter of 3.76 mm. For *D. mawsoni* , Parker et al. (2021) estimated that eggs of average density can rise from 1500 m to the surface in approximately 4–5 days. On this basis, we imposed a 1‐week ascent phase after spawning, during which eggs were assumed to rise to the surface without horizontal displacement. This simplification is consistent with the sensitivity analysis of Mori et al. (2016), who reported that allowing horizontal displacement during egg ascent had minor effects on final particle positions. This vertical ascent was not dynamically simulated and the extrapolation of egg‐buoyancy characteristics from *D. mawsoni* to *D. eleginoides* is an explicit biological assumption of our model.

Horizontal dispersal was then simulated using a strictly two‐dimensional (2‐D) Lagrangian framework. At the end of the assumed 1‐week ascent, particles were initialized at the ocean surface (z = 0 m; the model has no vertical coordinate) and advected for 18 weeks, corresponding to the pelagic larval duration (PLD) (North 2002; Lee et al. 2024). Vertical velocities, turbulent vertical mixing, and vertical shear in horizontal currents were not represented. We therefore assumed that satellite‐derived surface geostrophic velocity provides a first‐order approximation of horizontal transport experienced by eggs and larvae within the upper mixed layer. Previous studies have validated the use of surface geostrophic currents to the study of the transport of tracers distributed within the surface mixed layer (Venables and Meredith 2009; d'Ovidio et al. 2015; Lehahn et al. 2007; Ardyna et al. 2019; Sergi et al. 2020). No fixed mixed layer depth was prescribed in the simulations; mixed layer depth is used here only to evaluate the domain of validity of this surface‐flow approximation. This approximation is best suited to individuals occupying near‐surface waters and becomes less representative for larvae located below the mixed layer. Available observations indicate that Patagonian toothfish larvae occur mainly in the upper water column, between 0 and 250 m and frequently near the surface (Evseenko et al. 1995; North 2002; Lee et al. 2024). No active vertical migration was imposed, as in Mori et al. (2016); previous Lagrangian studies have likewise treated toothfish eggs and early larvae as passive particles transported in the surface layer (Mori et al. 2016, 2022).

We used surface geostrophic velocities derived from a multisatellite altimetry product (SEALEVEL_GLO_PHY_L4_MY_008_047, doi: https://doi.org/10.48670/moi‐00148) maintained by the CNES‐CLS operational system DUACS (Data Unification and Altimeter Combination System, https://duacs.cls.fr/). This product has spatial and temporal resolutions of, respectively, 0.125° (approximately 14 km in latitude and 9 km in longitude) and 1 day. The use of DUACS to study mesoscale transport pathways has been validated in the study region by previous studies (d'Ovidio et al. 2015; Nalivaev et al. 2026). Geostrophic velocities were extracted over the period 2000–2023.

A Lagrangian experiment of particle advection was performed using the LAMTA software (LAgrangian Manifolds Tracking Algorithm, module of the SPASSO package, v2.0) (Rousselet et al. 2025). Forward trajectories were derived using a fourth‐order Runge–Kutta scheme with a 6‐h timestep. Spawning cohorts were defined weekly between Weeks 22 and 30 of each year. Accounting for the imposed 1‐week ascent, numerical releases at the surface occurred 1 week later, on the Thursday of Weeks 23–31. Each surface release was then tracked for 18 weeks. Across the 2000–2023 simulations, this design produced an overall horizontal‐advection season extending from early June to mid‐December (approximately 7 June–12 December). Release locations were defined on a regular grid with a spacing of 0.05° in both latitude and longitude, corresponding to grid cells of approximately 5 km (latitude) by 3 km (longitude) at the latitude of Kerguelen. At each weekly release, 2641 particles were released in total: one from the centre of each 0.05° grid cell across the spawning area. When a particle first reached the coast (seabed depth ≥ 0 m), its horizontal position was held there for the remainder of the simulation.

To account for spatial variability in reproductive output, each particle was assigned a weight corresponding to the predicted egg production of the nearest 10 × 10 km grid cell at its release location. This approach allowed us to account for spatial variability in spawning when simulating larval dispersion and estimating larval retention rates. Hereafter, “particles” refer to egg‐weighted particles.

### 2.4 Dispersal Pathways

To identify the main dispersal pathways, we aggregated all particle positions across the entire pelagic larval duration (PLD) onto a common regular grid. Density heatmaps therefore represent time‐integrated trajectory occupancy (i.e., cumulative space use over the full PLD). These maps were used to visually assess the main dispersal pathways from the release ground. In addition to these density heatmaps, we derived a set of quantitative metrics describing particle transport behavior (e.g., residence patterns, pathway occupation and retention‐related indicators, see Appendix S1.6), allowing differences in the physical transport of larvae—driven by interannual variability in ocean circulation—to be compared across years. These analyses were used to assess how the relative contribution and balance among different dispersal pathways can explain the larval retention rates. Dispersal pathways were further contrasted between low‐ and high‐retention years, defined, respectively as years below the 10th percentile and above the 90th percentile of the time series of annual larval retention rate (2000–2023).

To relate larval dispersal pathways to regional circulation, we focused on the surface circulation definition of the Polar Front. Because larval dispersal was simulated using satellite‐derived surface geostrophic currents, we used a circulation‐based representation of the PF, based on altimetry data, rather than a hydrographic definition based on the location of Winter Waters. Following Park et al. (2019), the PF was represented by a geostrophic streamline constrained by its known pathway around Kerguelen. An annual PF streamline was identified for each year from seasonally averaged DUACS absolute dynamic topography, while preserving the topographically constrained pathway south and east of Kerguelen Island. Full details of the streamline construction are provided in Appendix S1.5.

PF‐associated jet intensity was calculated as the mean surface geostrophic current speed along the annual PF streamline within 67°–72° E and 48°–51° S (Figure S1). Pearson correlations were then used to test whether the annual proportion of particles transported through each dispersal pathway was related to PF‐associated jet intensity. We also examined the Subantarctic Front (SAF), which flows north of the plateau. An annual SAF streamline was identified with the same approach as for the PF (Park et al. 2019), without bathymetric constraint since the SAF flows in deep water about 350 km north of Kerguelen Island (Appendix S1.5.4). SAF intensity was calculated as the mean surface geostrophic current speed along the annual SAF streamline within 63°–73° E and 44°–47° S (Figure S1), and was related to larval retention in the same way as the PF‐associated jet intensity.

### 2.5 Temporal Variability in Larval Retention Rate

Because larvae have limited active swimming capacity at settlement, we assumed that settlement occurs in the immediate vicinity of the habitat occupied by age‐1 to age‐2 juveniles. On this basis, suitable recruitment areas were defined as areas located at depths between 0 and 150 m and in Skiff Bank (0–300 m), representing the areas where age‐1 and age‐2 juveniles are caught during the scientific bottom‐trawl surveys (unpublished data). At the end of the 18‐week simulation, all particles that were in the defined suitable recruitment areas were counted to obtain a measure of larval abundance. Larval retention success was calculated as the ratio of larval abundance in suitable recruitment areas to the total number of particles initially released from the spawning area (Appendix S1.7).

Larval retention temporal variability was assessed across all combinations of week/year of particle releases to characterize both intra‐seasonal (weekly) and inter‐annual variations in larval retention. Sensitivity to recruitment depth and PLD were tested to evaluate the robustness of the larval retention rate metric. Specifically, we tested the impact of alternative recruitment depth thresholds (0–200 m, 0–300 m, 0–500 m, 0–700 m, and 0–1000 m) as well as shorter PLD for simulations (10 and 15 weeks). The resulting larval retention rates were compared to those obtained under the reference scenario (0–150 m, 18 weeks) and presented in Figures S3 and S4.

### 2.6 Spatial Variability in Larval Retention

Spatial variability of larval retention was examined by mapping the distribution of particles at the end of the simulation period. Kernel density estimation of particles was performed using the ggtern package (Hamilton 2024) (function kde2d.weighted), enabling the identification of larval hotspots after dispersal for each year of simulations. For inter‐annual comparisons, we restricted the analysis to simulations initiated in weeks 24–28 when the spawning activity is highest (designated as the spawning peak) (Ouzoulias et al. 2025).

To identify the spawning areas that contribute most to larval retention, as well as the recruitment areas that are most successful in a given year, we divided the spawning region into three subareas: North, West, and South. The northern subarea includes shallower spawning grounds, the western subarea corresponds to the main spawning hotspot active in June–July, and the southern subarea represents a marginal spawning area with limited activity later in the season (August–September) (Ouzoulias et al. 2025). The recruitment zone (defined as the 0–150 m depth layer) was divided into five subareas: North‐West (NW), West, North‐East (NE), South and Skiff bank (0–300 m) (Figure 3). These five subareas encompass the principal Kerguelen shelf headlands and adjoining shelf sectors.

We first evaluated the contribution of each spawning zone to the overall larval retention (in %) by assigning to each particle its subarea of release. Contributions were expressed annually as the percentage of total released eggs originating from each spawning subarea and reaching any suitable recruitment habitat compared to the total number of particles released (Appendix S1.7).

Then, we assessed the contribution of each recruitment zone to the overall larval retention (in %) by assigning to each particle its subarea of recruitment. Contributions were expressed annually as the percentage of total released eggs reaching each recruitment subarea compared to the total number of particles released (Appendix S1.7).

### 2.7 Relationship Between Jet Intensities and Larval Retention

We used Gaussian generalized linear models (GLMs) to assess the relationship between annual larval retention ($R_{y}$) and standardized jet intensity indices ($z\left(I_{y,f}\right)$). Annual retention was expressed as the total number of particles successfully retained within suitable recruitment areas. Letting $I_{y,f}$ denote the annual intensity index for year y and jet f (PF or SAF), and $z\left(I_{y,f}\right)$ its standardized version, we fitted, for PF and SAF separately, a Gaussian GLM with a log‐transformed response and an identity link function:

(1) $$\log\left(R_{y}\right)=\alpha +\beta \;z\left(I_{y,f}\right)+\varepsilon_{y},\;\;\varepsilon_{y}∼N\left(0,\sigma^{2}\right),$$

where $\varepsilon_{y}$ denotes the residual error for year y, assumed to be independently and identically distributed with mean zero and variance $\sigma^{2}$.

## 3 Results

### 3.1 Spatial Distribution of Eggs Produced

The model of female spawners' relative abundance provided a good fit to the observed data (Table S1). High abundances were predicted west of Kerguelen Island, predominantly at depths between 1500 and 1800 m (Figure 4A). The model of female spawner length also provided a good fit to the observed data (Table S2). The prediction map of female spawner's mean total length across the western region of the Kerguelen EEZ revealed that deep regions were predicted to host larger mature females (> 100 cm), while smaller mature females (< 100 cm) were predicted on shallower areas, between 500 and 700 m (Figure 4B).

**FIGURE 4:**
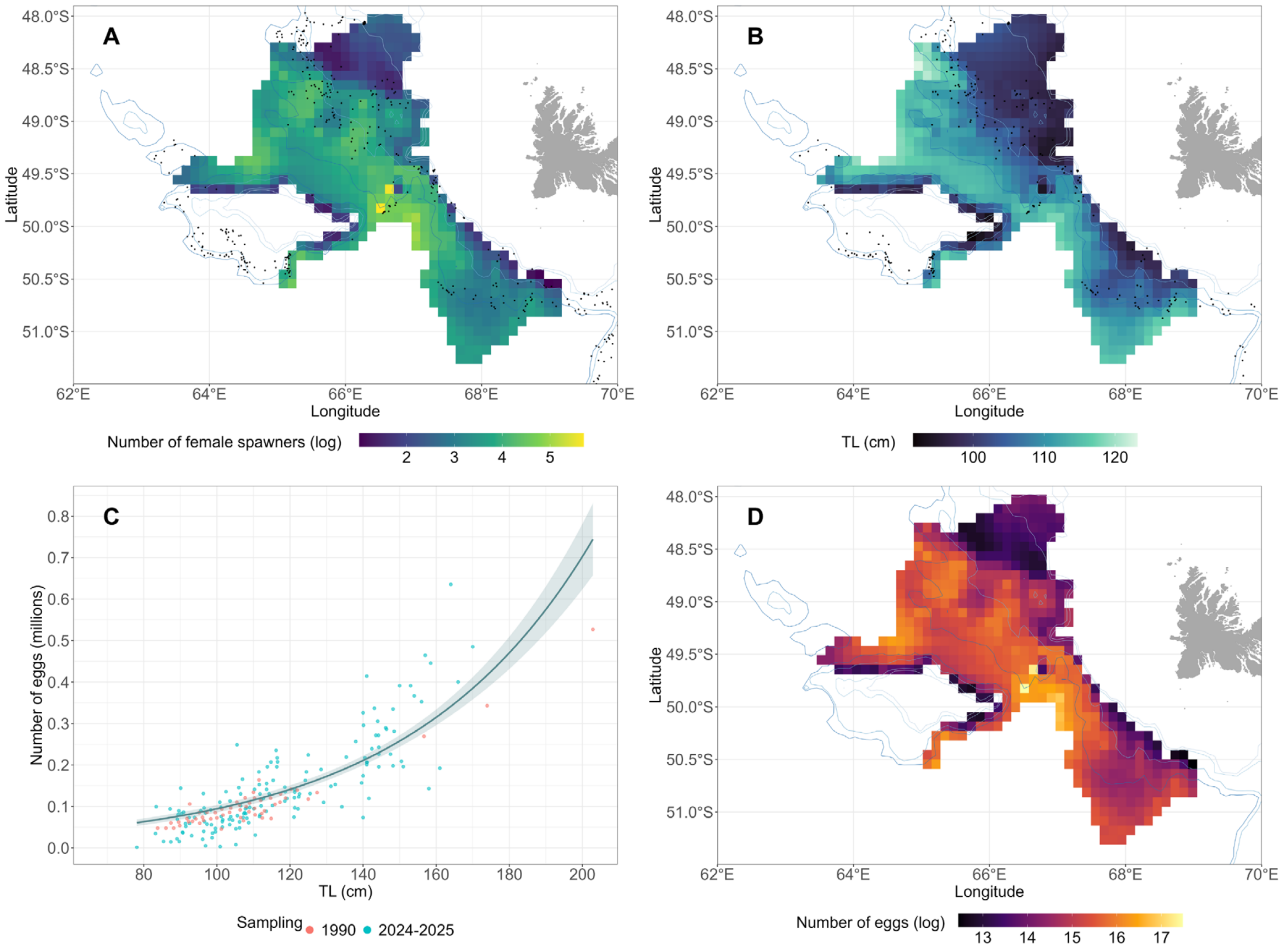
(A) Spatial predictions of female spawner abundance (log‐scaled), (B) Spatial predictions of average total length, (C) Fecundity‐length relationship with the two data sources, and (D) Spatial predictions of total egg production. The blue lines on the maps correspond to the 500, 700, 1500 and 1800 m isobaths. The small black dots correspond to lines observed for biological sampling, from 2020 to 2024.

The fecundity was exponential, ranging from 1527 to 635,514 eggs per female depending on their body size (Figure 4C).

The reproductive outputs map revealed a hotspot of egg production around 50° S and 67° E and between 1500 and 1800 m. In the spawning hotspot, estimated egg production ranged from 0.265 to 40.376 million eggs per cell. Average total length (TL) had a minor effect on the total egg production (Figure 4C) because of the range of the predicted TL (91–123 cm). Total egg production was mostly influenced by the female spawner relative abundance.

### 3.2 Larval Dispersal Pathways

The density heatmaps revealed several recurrent dispersal pathways around the Kerguelen Plateau (Figure 5). A major pathway extended southward and eastward from the spawning grounds and closely followed the PF streamline. The number of particles crossing the southern corridor of this pathway was strongly and positively correlated with PF‐associated jet intensity (r=0.75, p<0.001; Figure S8). This pathway was associated with long‐distance export away from the shelf, with many particles ending east of Kerguelen Island and beyond shallow recruitment areas. Low‐retention years were characterized by a greater contribution of this southern pathway along the PF streamline.

**FIGURE 5:**
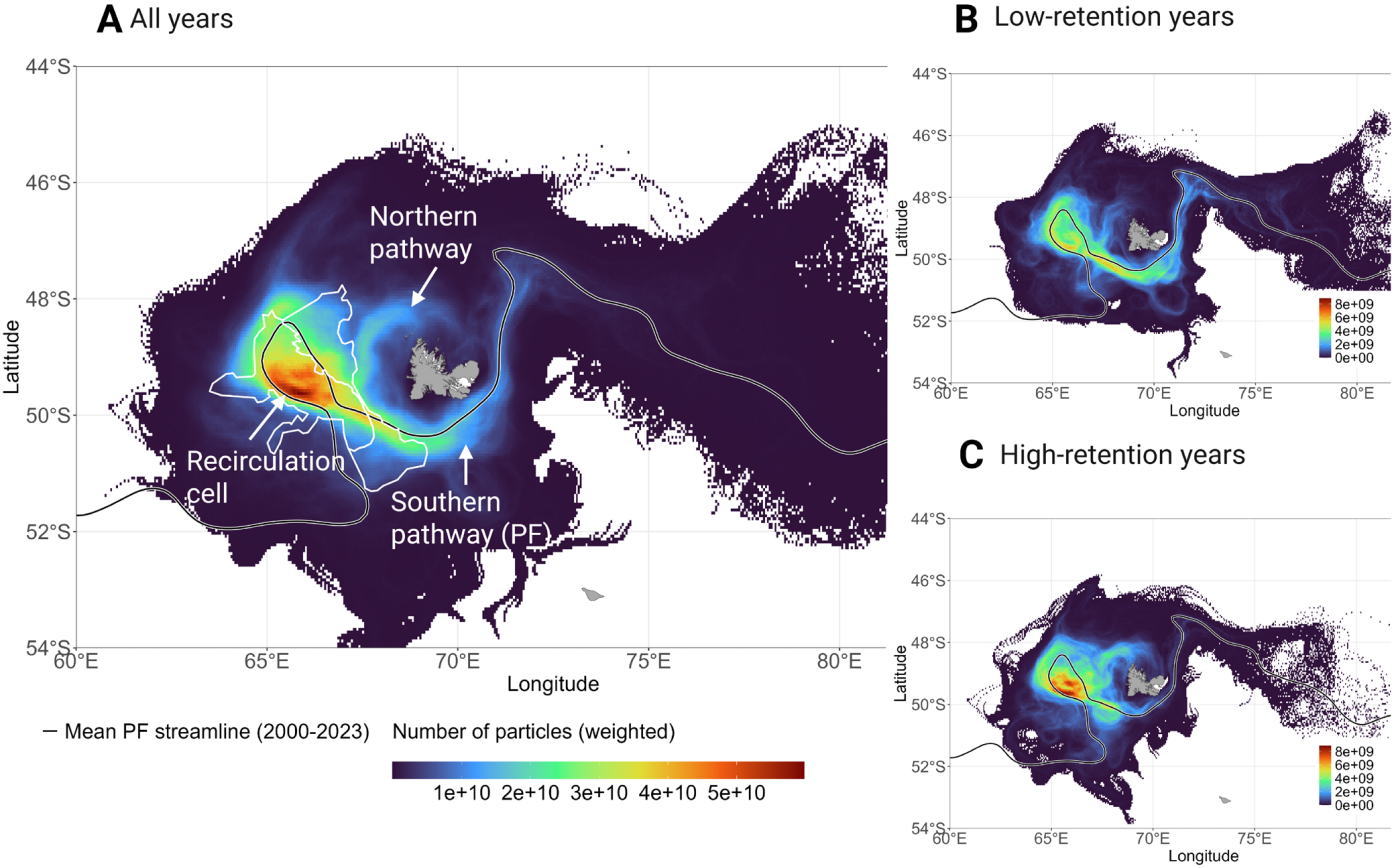
Larval dispersal pathways during the 18‐week simulation: All years (A), low (B) and high (C) retention years. The release area is represented in white.

A second pathway occurred north of the island, along the northern shelf and slope. Annual transport through the NW corridor was negatively correlated with PF‐associated jet intensity (r=−0.55, p=0.005; Figure S8) and with transport through the southern PF‐associated corridor (r=−0.84, p<0.001; Figure S8). Transport through the NW corridor was also positively correlated with larval retention (r=0.60, p=0.002; Figure S8), indicating that years with greater use of the northern pathway tended to have higher retention.

A third pathway corresponded to a localized recirculation cell west of Kerguelen Island, near the spawning area. This pathway was characterized by short dispersal distances and prolonged residence near the release area. The proportion of particles remaining within this region was negatively correlated with the number of particles transported along the southern PF streamline pathway (r=−0.66, p<0.001). Most particles eventually left the recirculation cell and joined either the northern shelf pathway or the southern PF streamline pathway.

### 3.3 Variability of Larval Retention Rate

We did not find a marked systematic effect of the spawning week on larval retention rate across the full spawning window (Figure S2), so the results hereafter focus on releases during Weeks 24–28, which correspond to the peak of the spawning period of Patagonian toothfish (Ouzoulias et al. 2025).

Overall, larval retention showed pronounced interannual variability over the study period (2000–2023), ranging from approximately 9% in 2023 to 28% in high‐retention years such as 2000, 2016, and 2022 (Figure 6). During the first decade of the time series, retention rates generally remained closer to the long‐term mean (19%), whereas the period after 2010 included more frequent low‐retention years, notably 2010, 2015, 2017, and 2023.

**FIGURE 6:**
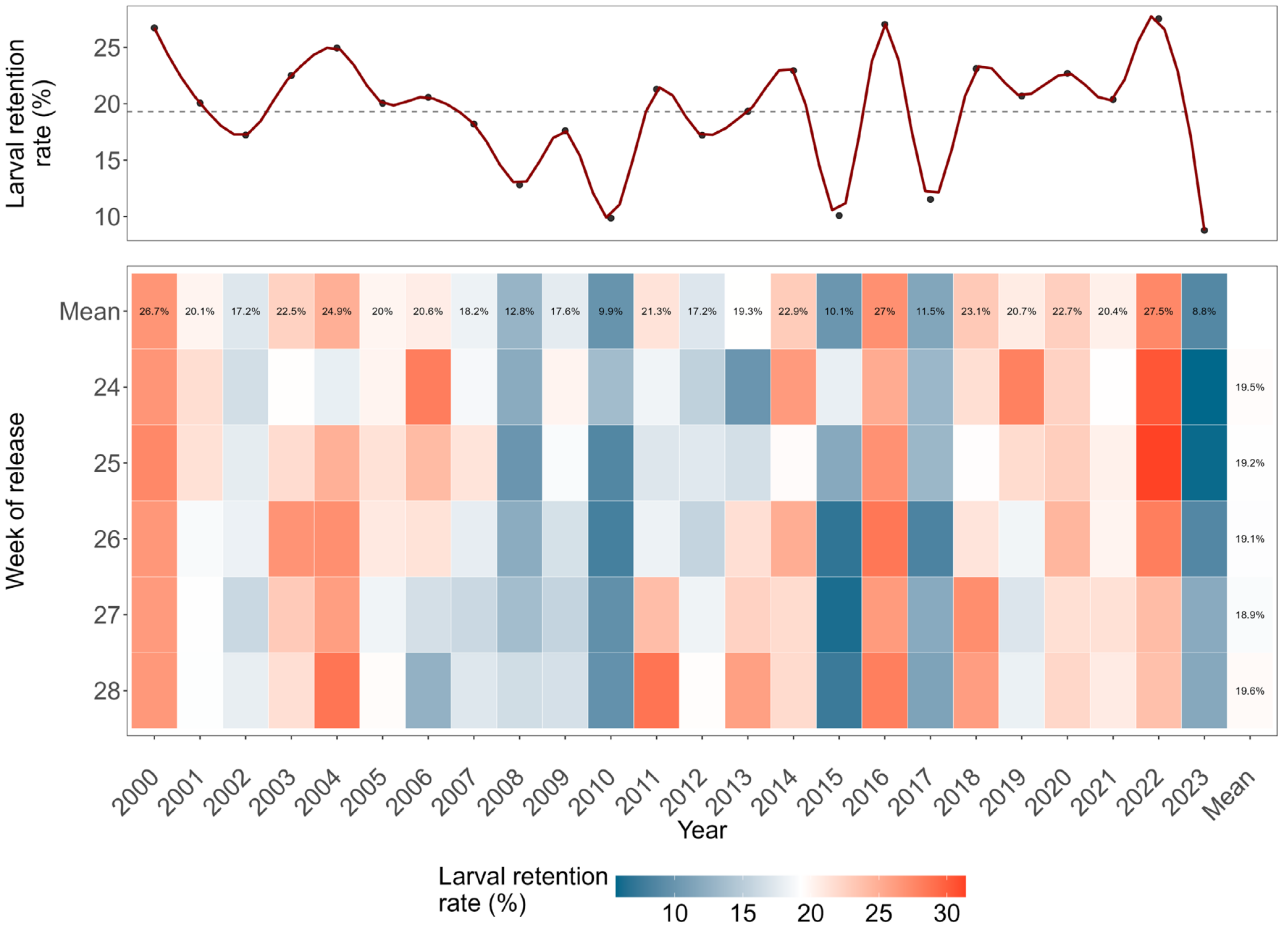
Larval retention rate from 2000 to 2023 across release Weeks 24–28. The top row (“Mean”) represents the inter‐annual mean larval retention rate averaged across Weeks 24–28, while the rightmost column represents the weekly mean retention rate averaged across all years. The upper panel displays annual larval retention rates together with a locally smoothed trend (red line).

Larval retention increased with simulation duration, with the 18‐week simulations generally yielding the highest values across years (Figure S3). About one third of the increase between 10 and 18 weeks was due to particles that had left recruitment habitat and later returned (more details in Appendix S1.6).

Retention rates also increased with the depth range considered suitable for settlement (Figure S4), reaching values of up to approximately 65% under the 0–1000 m scenario. Relaxing the depth threshold enlarges the sea‐floor area classified as suitable, so a greater proportion of particles end their drift over suitable area. Importantly, despite substantial differences in absolute retention values, there was no modification of the relative inter‐annual patterns of larval retention rate.

#### 3.3.1 Identification of the Recruitment Areas

Overall, Kernel density distributions revealed that larval densities were concentrated on the main headlands of the Kerguelen Island (Figure 7), highlighting recurrent retention hotspots near the shelf. Annual maps of larval density at the end of the simulation period revealed substantial inter‐annual variability in spatial retention patterns (Figure S5). In some years, larval retention was strongly localized over the shelf or within specific coastal retention zones (e.g., 2000, 2014, 2016, and 2018), whereas in other years larvae were more diffusely distributed, with very low densities in shallow areas (e.g., 2010, 2012, and 2015). Retention in the north‐west recruitment area was negatively correlated with PF‐associated jet intensity (r=−0.48 and p=0.019), with higher retention on the northern shelf occurring in years characterized by weaker PF‐associated circulation. Retention in Skiff Bank was consistently low.

**FIGURE 7:**
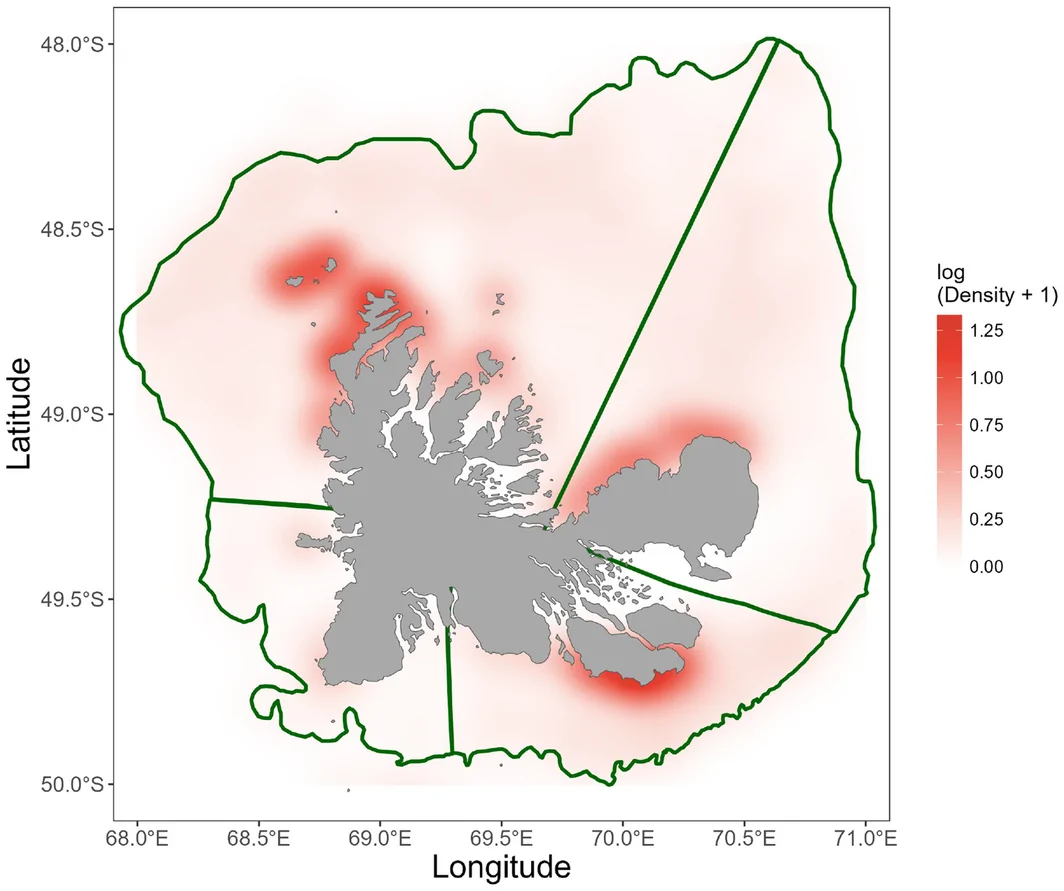
Spatial distribution of larval density at the end of the dispersal simulation: Kernel density estimates of particle positions after 18 weeks of advection, aggregated across all simulation years and for releases during Weeks 24–28. Green lines delineate the sub‐recruitment areas (depth ≤ 150 m). Skiff Bank is excluded.

#### 3.3.2 Contribution of Recruitment and Spawning Subareas in the Overall Larval Retention Rate

Larval retention rate varied substantially among recruitment zones over the study period (2000–2023, Figure 8A).

**FIGURE 8:**
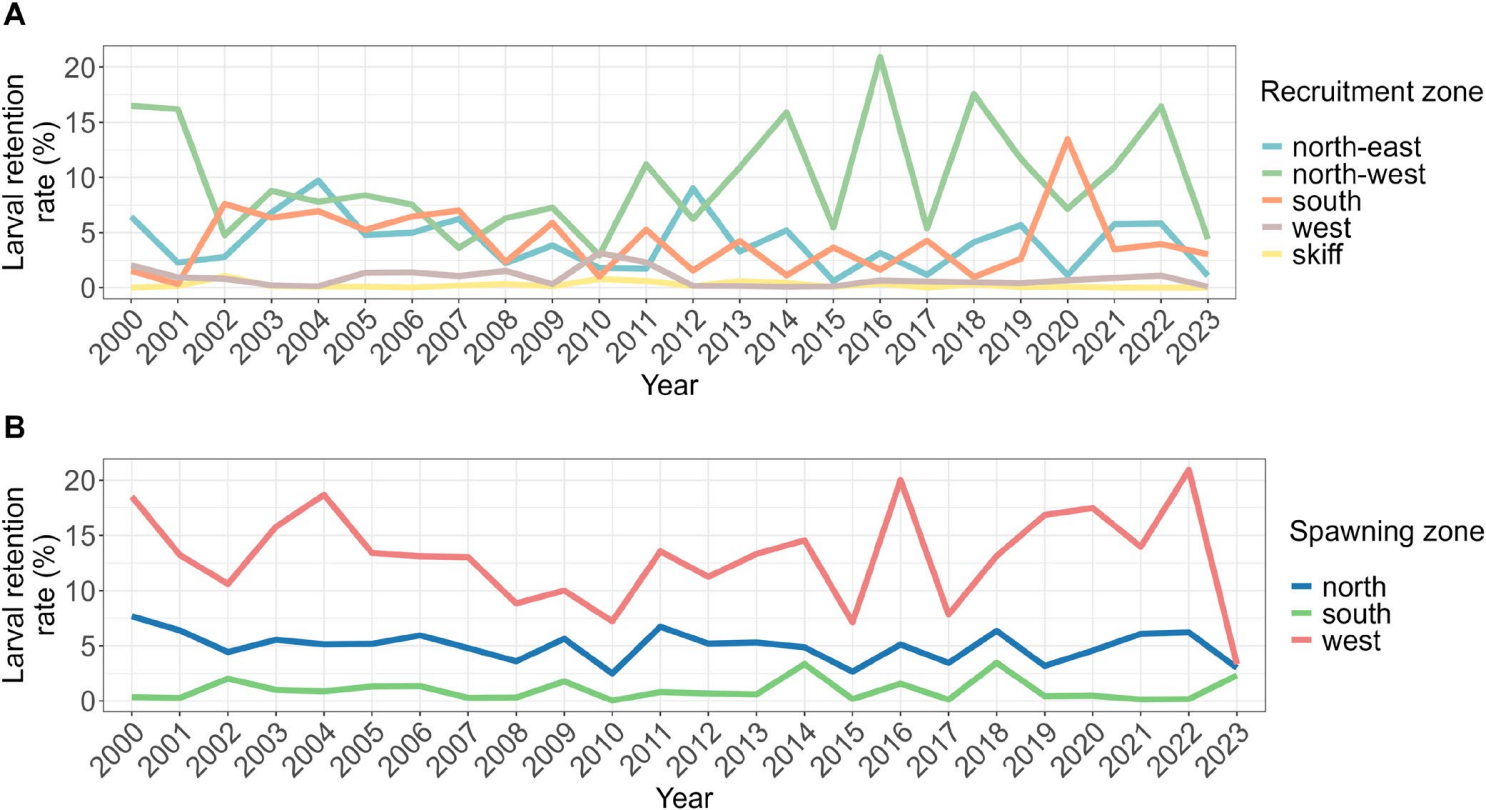
Spatial distribution of larval retention by recruitment area (A) and contribution of each spawning area to the overall retention (B) (in %).

The larval retention rate in the NW recruitment area was the highest but inter‐annual variation was high, with several marked peaks, notably in 2014, 2016, 2018 and 2022, when retention locally reached 16%–21%. The NE zone contributed at intermediate levels, generally between 2% and 8%, with episodic peaks (e.g., 2004 and 2012). The South recruitment zone displayed strong year‐to‐year variability, alternating between low contributions and occasional high values, particularly from 2002 to 2007 (5%–8%) and in 2020, when retention temporarily exceeded 10%. In contrast, the west area and the Skiff area consistently exhibited very low retention rates (generally < 2%), indicating a minor contribution to overall larval retention. A marked change in the relative contributions of recruitment areas was apparent around 2010. From 2002 to 2009, contributions from the NW, NE and South recruitment zones were of comparable magnitude, with no single zone clearly dominating. After 2010, larval retention rates in the NE, South and West areas were generally lower, while the NW recruitment zone accounted for a larger share of overall retention.

Larval retention also differed markedly among spawning zones (Figure 8B). The western spawning area overwhelmingly dominated larval contributions throughout the study period. The northern spawning zone provided a moderate but persistent contribution, generally around 3%–7%, with limited inter‐annual variability. In contrast, the southern spawning zone contributed only marginally to larval retention, with values usually below 2% and sporadic increases in a few years (e.g., 2014 and 2018).

Connectivity differed markedly among spawning areas (Figure S7). Despite accounting for only 10% of the eggs released, the northern spawning area had the highest retention rate: 50% of its eggs reached a recruitment area, compared with 17% for the western spawning area and 8% for the southern spawning area. The strongest connection from the northern spawning area was to the NW recruitment area, which received 29% of the eggs released there.

### 3.4 Relationship Between Jet Intensities and Larval Retention

Annual larval retention was negatively related to PF‐associated jet intensity (Figure 9; Table S3). The Gaussian GLM fitted to log‐transformed annual retention showed that years with stronger surface geostrophic flow along the annual PF streamline had fewer retained larvae. The model explained 24% of the interannual variability in larval retention.

**FIGURE 9:**
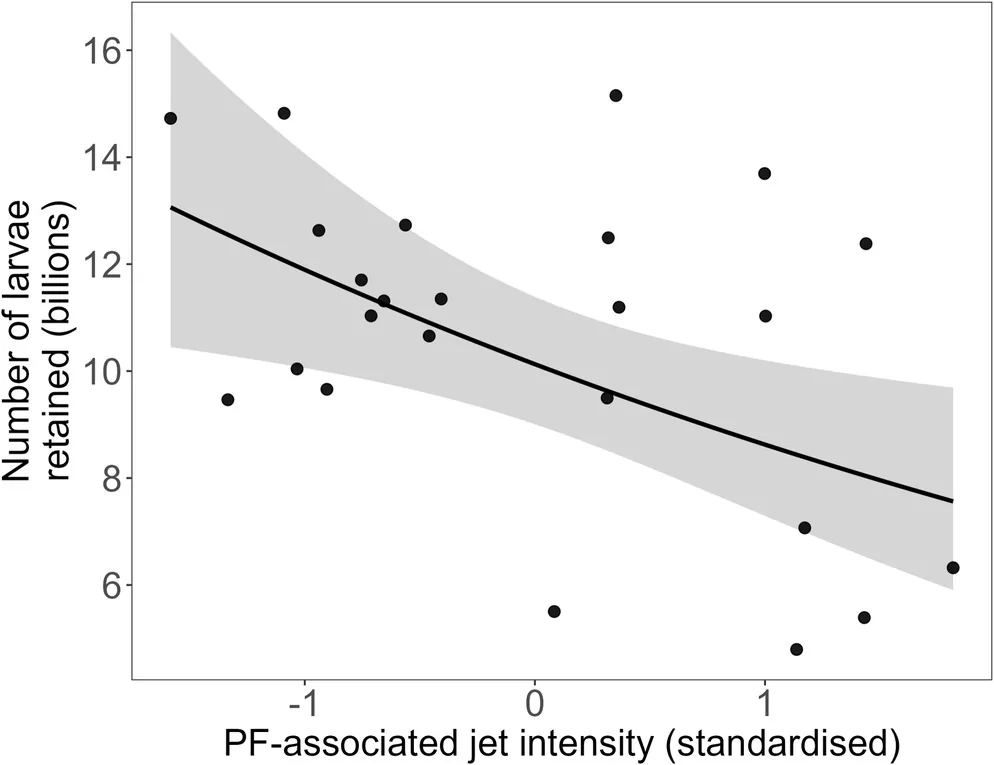
Modeled relationship between PF‐associated jet intensity and annual larval retention.

Some years, for example, 2003 and 2004, departed from this general relationship. In these years, despite relatively high PF‐associated jet intensity, larval retention remained high because the southern PF‐associated pathway curved back toward shallow shelf areas east of Kerguelen Island, allowing particles to reach suitable recruitment habitat (Figure S6).

No significant relationship was detected between larval retention and the intensity of the Subantarctic Front (SAF) (Table S4). However, SAF intensity was positively correlated with retention in the NE area (r=0.63, p=0.001, Figure S8).

## 4 Discussion

The PF emerged from our results as a key element explaining larval retention variability: years with stronger flow along the annual PF streamline were characterized by greater eastward transport south and east of Kerguelen Island and lower retention on the shelf, with more larvae advected away from the shelf toward the eastern part of the plateau. Between 2000 and 2023, larval retention rate, following the PF‐associated jet intensity, varied from 9% to 28%. The latter part of the time series, particularly after 2010, was characterized by stronger year‐to‐year fluctuations, a greater contribution of the NW recruitment area, an average lower retention in the South and NE, and more frequent low‐retention years. Our findings also underscore the value of an integrated modeling framework coupling spawning dynamics and ocean circulation. Building on new findings on toothfish spawning dynamics and long‐term altimetry data, we showed for the first time that there is the potential for self‐recruitment in the northern part of the Kerguelen Plateau.

### 4.1 Role of the PF Streamline in Larval Retention

Our results show a strong association between larval retention and the surface circulation associated with the PF. Years with stronger flow along the annual PF streamline were characterized by greater eastward transport south and east of Kerguelen Island and lower retention on the shelf. In contrast, years with weaker PF‐associated circulation showed greater transport along the northern shelf pathways and higher larval retention. Southern Ocean fronts are hydrographic boundaries that are often associated with strong geostrophic jets, although the two features are not necessarily overlapping (Chapman et al. 2020). In the present study, the relationship with larval retention was therefore quantified from the strength of the PF‐associated jet rather than from the hydrographic properties of the front.

Average annual larval retention varied widely over the study period, from approximately 9% in 2023 to 28% in high‐retention years, and extreme low‐retention years occurred more frequently after 2010. This pattern is consistent with evidence that mesoscale current variability has intensified over recent decades in eddy‐rich regions of the Southern Ocean, with increases up to 5% per decade (Martínez‐Moreno et al. 2021).

The annual PF streamline showed relatively limited interannual displacement around Kerguelen (Figure S6), particularly south and east of the island where its pathway is strongly constrained by bathymetry. A different component of PF variability is captured by its hydrographic definition (i.e., the northernmost extent of Antarctic Winter Waters (AWW) (Chapman et al. 2020)). Around Kerguelen, this hydrographic boundary can show substantial seasonal and interannual variability, particularly west of the plateau where bathymetric constraints are weaker (Pauthenet et al. 2018). The AWW distribution can also respond rapidly to anomalously warm conditions. Azarian et al. (2024) showed that marine heatwaves in the Southern Indian Ocean are associated with a reduced probability of AWW presence and a southward displacement of this water mass. Under future warming, AWW are projected to shift southward, although the magnitude of this shift varies spatially and among climate models (Azarian et al. 2024). These two components of PF variability may affect larvae through different mechanisms. Variability in the PF‐associated geostrophic jet directly modifies horizontal transport and therefore larval dispersal pathways. In contrast, variability in the hydrographic PF may alter the temperature and water‐mass conditions experienced by larvae during dispersal. Our simulations resolve the first mechanism through annually varying surface geostrophic circulation, focusing on the dispersal patterns of the larvae.

In contrast to the PF‐associated jet, SAF intensity was not significantly related to total larval retention. SAF intensity was, however, positively correlated with retention in the NE recruitment area. Given that the SAF lies well north of the main dispersal pathways around Kerguelen, we interpret this relationship cautiously as an association with broader regional circulation rather than as evidence of a direct effect on larval transport.

At larger scales, the Southern Annular Mode (SAM) provides a useful description of atmospheric variability affecting Southern Ocean circulation. SAM represents the dominant mode of atmospheric variability in the extratropical Southern Hemisphere and reflects changes in both the position and intensity of the circumpolar westerly wind belt (Rogers and van Loon 1982; Thompson and Solomon 2002). Positive SAM phases are characterized by strengthened and poleward‐shifted westerly winds, which are associated with changes in the Antarctic Circumpolar Current and its frontal circulation (Fogt and Marshall 2020; Su et al. 2021). Over the last 50 years, studies indicate a trend toward more positive SAM phases, corresponding to stronger circumpolar westerly winds (Meredith and Hogg 2006; Böning et al. 2008; Fogt and Marshall 2020). In our analysis, the annual SAM index was not significantly related to larval retention (r=−0.29, p=0.18), whereas the mean SAM index during austral winter (June–August) showed a weak negative correlation with retention (r=−0.43, p=0.035). The mean SAM indices for austral winter (June–August) and spring (September–November) were both positively correlated with PF‐associated jet intensity (JJA: r=0.46, p=0.024; SON: r=0.46, p=0.022; see Appendix S1.6 for details on the SAM indices and Figure S8 for the full correlation matrix). These pairwise relationships are consistent with a possible indirect pathway linking large‐scale atmospheric variability to larval retention through changes in regional circulation, but they do not constitute a formal mediation test and should not be interpreted as demonstrating causality. The tendency toward more positive SAM conditions therefore provides a relevant climatic context for the observed variability in frontal circulation and larval retention. Projections of future SAM evolution remain uncertain. Coupled chemistry–climate models suggest that the recovery of the ozone hole may drive the SAM toward more negative values during austral summer, whereas general circulation models indicate that increasing greenhouse gas concentrations are likely to continue reinforcing the positive phase of the SAM in other seasons (Watson et al. 2012; Fogt and Marshall 2020). These opposing forcings, acting at different times of the year, generate substantial uncertainty regarding the magnitude and timing of future SAM changes, and consequently the future variability of Southern Ocean frontal circulation. Future changes in SAM could therefore modify the circulation conditions associated with larval transport and retention, but the direction and magnitude of this effect cannot be inferred from the present analysis.

Variation in PF‐associated circulation was also associated with changes in larval dispersal pathways, final spatial distributions, and the relative contributions of spawning grounds to overall larval retention.

### 4.2 Influence of PF‐Associated Circulation on Connectivity Between Spawning and Recruitment Areas

#### 4.2.1 Spatial and Demographic Drivers of Spawning Areas Contributions to Larval Retention

The western spawning area contributed the most to the total number of larvae reaching recruitment areas, primarily because it is larger and encompasses the main spawning hotspots identified by Ouzoulias et al. (2025). Inter‐annual variations in spawning distribution and egg production were not accounted for due to a lack of data. We therefore assumed that spawning dynamics (distribution, timing and egg production) described in Ouzoulias et al. (2025) remained stable from 2000 to 2023. On the southern Kerguelen Plateau (HIMI), spawning‐site fidelity likewise remains undetermined: studies identified key hotspots but did not assess inter‐annual variations (Welsford et al. 2012; Yates et al. 2018). In contrast, in South Georgia, Bamford et al. (2024) studied inter‐annual variation in spawning dynamics (1997–2021) and found major stable hotspots and secondary sites with minor shifts in location. More consistent winter sampling and continued application of fecundity protocols should enable the estimation of spawning‐site fidelity and year‐to‐year changes in spawning effort, which may influence larval retention. Total egg production depends on the size structure of the spawning female stock: fecundity of Patagonian toothfish increases steeply with body size, so that large, older females contribute disproportionately to egg production relative to smaller, potentially first‐time spawners. In our study, we did not consider temporal changes in the size of mature females over 2000–2023. This assumption should not affect larval retention rates (expressed here as relative metrics), but it likely affects absolute larval abundance estimates.

Although the western spawning ground dominated overall contributions because it encompasses the main spawning hotspots, in some years, a large fraction of eggs released in the west were trapped locally and did not reach shelf recruitment areas. By contrast, although spawning effort is lower in the northern sector, eggs released there were more frequently advected toward favorable shallow habitats on the northern shelf. Accordingly, the strongest connectivity occurred between the northern spawning grounds and the NW recruitment zone. Conversely, larvae from the southern spawning ground were rarely retained.

#### 4.2.2 Influence of PF‐Associated Circulation on the Contribution of Main Recruitment Areas

The depth at which Patagonian toothfish larvae settle remains unknown. Juveniles aged 1 year are mostly found in nearshore waters and on the top of submarine banks. Given the limited swimming capacity of larvae at settlement, we assumed that settlement occurs close to the habitat of age‐1 to age‐2 fish. Shelf surveys have repeatedly reported high densities of age‐1 to age‐3 juveniles on the northern shelf (Duhamel et al. 2019; Péron et al. 2025), supporting the definition of these areas as suitable nurseries. Although DUACS altimetry provides long and consistent time series, its reliability is reduced in coastal waters (Nalivaev et al. 2026). We therefore defined larval retention as the arrival of particles within shallow shelf habitats (0–150 m) and Skiff Bank. Sensitivity analyses showed that increasing the depth range of suitable recruitment areas increased retention rates but did not modify their inter‐annual variability (Figure S4). Our results are consistent with previous simulations identifying the northern shelf as a recurrent retention area for larvae released west of Kerguelen Island (Mori et al. 2016). Overall, the NW and NE recruitment areas contributed the most to larval retention, largely because they cover extensive shallow shelf surfaces associated with a softer slope in the northern part of the plateau. Larval retention rate varied substantially among recruitment zones over the study period. From 2002 to 2009, contributions from the NW, NE, and South recruitment zones were of comparable magnitude, with no single zone clearly dominating. Retention in NW recruitment zones was generally higher during years characterized by weaker PF‐associated jet intensity (Figure S8), when transport along the northern shelf pathway was enhanced. After 2010, larval retention rates in the NE, South, and West areas declined, and the NW recruitment zone dominated larval retention, accounting for most high‐retention years. The increasing predominance of the NW shelf as a recruitment area after 2010 reflects the decline in larval retention in the NE and South sectors. By concentrating retention within a limited area, the population becomes increasingly dependent on local processes, thereby reducing its overall resilience and increasing its sensitivity to other factors affecting the post‐dispersal phase.

### 4.3 Limitations of the Two‐Dimensional Dispersal Framework

In our two‐dimensional dispersal framework, trajectories advected by surface geostrophic currents are used as a proxy for larval dispersal. This approximation is most relevant for larvae occupying the near‐surface part of the water column. Patagonian toothfish larvae have indeed frequently been observed near the surface (North 2002); for example, at South Georgia, 23 of 43 pelagic larval and early juvenile specimens examined by North (2002) were collected in the upper 3 m. However, larvae have also been reported at depths down to approximately 250 m. Around Kerguelen, mixed‐layer depth varies strongly with season and location (see Table S5). As the mixed layer becomes shallower in spring, larvae at greater depths may experience currents that differ from the surface geostrophic currents used here.

A second uncertainty concerns the initial ascent of eggs. Because direct buoyancy measurements are unavailable for Patagonian toothfish, the assumed one‐week ascent from the spawning depth to the surface is based on measurements from Antarctic toothfish *D. mawsoni* (Parker et al. 2021). Potential interspecific differences in egg density, buoyancy, developmental rate, and the effects of vertical currents or turbulence could alter both ascent time and the horizontal position at which eggs enter the surface layer. These processes are not represented in the present 2‐D framework and should be addressed when species‐specific observations or three‐dimensional circulation products become available.

### 4.4 From Dispersal Pathways to Recruitment Drivers

#### 4.4.1 Effects of Environmental Factors on Recruitment

Several environmental drivers are expected to influence early life stages and thus recruitment in the Southern Ocean. There is strong evidence that significant and large‐scale changes are underway in this region (Constable et al. 2014; Auger et al. 2021; Van de Putte et al. 2021), which is among the areas of greatest oceanic heat uptake globally (Garcia‐Soto et al. 2021). The mean surface warming from 1982 to 2019 has been estimated at 0.03°C per decade in the Southern Indian Ocean (Azarian et al. 2023) (70°–30° S, 20°–120° E).

Beyond long‐term trends, extreme events such as marine heatwaves are projected to increase in frequency and intensity. Marine heatwaves are defined as prolonged periods of anomalously warm water exceeding a given threshold for at least five days (Hobday et al. 2016). North of the Kerguelen Plateau, higher marine heatwave intensities have already been detected, and future projections identify the region as a hotspot for marine heatwave activity (Azarian et al. 2023). Long‐term climate trends and extreme events in the Southern Ocean may directly influence recruitment by affecting the early life stages, as temperature strongly constrains embryonic development, egg survival, larval growth, size at hatching, and PLD through physiological processes (Morales et al. 2024; Lechner et al. 2025). Young et al. (2018) showed that ocean warming led to shorter PLD but higher cumulative egg and larval mortality in Antarctic fishes, highlighting potential effects of warming on dispersal and survival. At South Georgia, high toothfish recruitment has been linked to cooler spawning temperatures (Belchier and Collins 2008), and juveniles around the Falkland Islands show age‐specific associations with environmental conditions (Lee et al. 2021).

Our integrated modeling framework could be further developed to incorporate environmental conditions experienced by larvae explicitly. Individual‐based modeling approaches have been successfully used to assess how environmental variability, such as temperature, interacts with ocean circulation to shape larval dispersal and settlement patterns (Hidalgo et al. 2011; Young et al. 2018). Applying such approaches to Patagonian toothfish represents a promising perspective, but currently remains limited by major knowledge gaps on early life stages, including PLD, size, thermal tolerance, and swimming capacity.

#### 4.4.2 Effect of Biotic Interactions on Recruitment

In this study, we focused on larval retention, but processes occurring after the larval retention are also critical for recruitment success.

Studies showed that climate‐driven shifts in larval prey quality and composition can affect recruitment variability (Siddon et al. 2013). For toothfish, although little is known about larval ecology, Koubbi et al. (2009) reported that notothenioid larvae feed mostly on small zooplankton such as ciliates, small copepods, and early euphausid stages. Although population trends of these zooplankton groups are unknown, they rely on primary production and phytoplankton dynamics. On the Kerguelen Plateau, larval development of several demersal fish species appears to be timed to coincide with planktonic blooms and peaks in zooplankton production (Koubbi et al. 2009), consistent with a match–mismatch framework. By analogy, the spawning season of Patagonian toothfish may also be synchronized with local plankton dynamics, facilitating larval development. Several phytoplankton blooms have been reported east of the Kerguelen Plateau, including intense offshore plumes (Blain et al. 2001), which may overlap with the retention areas we identified, particularly in the NE. Beyond bloom location, previous studies documented inter‐annual variability in phytoplankton spatial patterns (Park et al. 2008) and showed that the position of the PF may influence annual blooms: Blain et al. (2007) demonstrated that the large phytoplankton bloom over the Kerguelen Plateau is sustained by natural iron fertilization linked to the interaction between the PF, the plateau bathymetry and vertical mixing, which controls the supply of iron‐rich deep waters to the surface. Inter‐annual shifts in the PF can therefore modulate the extent, timing, and intensity of the bloom by altering iron supply and nutrient refilling over the plateau. Such variability could create mismatches between larvae and the bloom on the Kerguelen Plateau, with potential consequences for larval survival and recruitment.

Climate‐driven changes may also influence recruitment through more indirect pathways. Variability in temperature, productivity, and circulation can affect fish recruitment by modifying adult populations, community structure, and population connectivity. At the scale of the Southern Ocean, climate‐driven variability in temperature, circulation, and sea ice has been shown to affect fish and squid populations, including connectivity (Caccavo et al. 2021). On the Kerguelen Plateau, long‐term surveys indicate marked changes in demersal fish assemblages since the early 2000s, partly associated with temperature‐related variables and large‐scale climate indices such as the SAM (Williams et al. 2025). However, these results primarily describe adult distributions and community‐level responses. The consequences of such ecological changes for the early life stages of Patagonian toothfish remain largely unknown, although they may influence recruitment through mechanisms including predation on eggs and larvae or competition for food resources or habitats.

Our results revealed a high larval supply in the NW shelf, but high retention rates do not necessarily translate into proportional numbers of juveniles if post‐settlement survival is constrained by density‐dependent processes. Recruitment success may therefore be limited by local carrying capacity: beyond a certain larval density, additional individuals may no longer survive or contribute significantly to recruitment (Houde 2008).

### 4.5 Implications for Fisheries Management

Years characterized by strong export along the Polar Front were associated with weak larval retention on the shelf, providing a physically based indicator of potentially unfavorable recruitment conditions. In this context, indices derived from PF‐associated jet intensity may offer useful early‐warning proxies for recruitment anomalies and help interpret past recruitment variability. These results also call for a re‐examination of how recruitment is represented in stock assessment models. Rather than assuming stationary recruitment processes with constant variance, our findings support the use of alternative recruitment scenarios that explicitly account for climate change and non‐stationary processes (Szuwalski and Hollowed 2016). Incorporating scenarios informed by physically based proxies, such as the PF‐associated jet intensity, would allow assessment frameworks to better capture the range of plausible recruitment outcomes under ongoing climate change and provide a more realistic basis for assessing population dynamics.

Our study further highlights the importance of the size structure of the spawning stock. Fecundity increases exponentially with female size, implying that large females contribute disproportionately to reproductive output. By selectively removing larger individuals, fishing may have altered the size distribution of mature females over recent decades, thereby reducing effective egg production. This consideration is likely to extend to other long‐lived species and provides a quantitative basis that can be used by fisheries managers to inform management strategies.

Our results highlight the spatial structure of early‐life connectivity on the Kerguelen Plateau. Larvae originating from spawning grounds within the French EEZ are predominantly retained over the northern shelf, suggesting self‐recruitment in this sector. However, our analysis was restricted to the northern part of the plateau because spawning areas have not yet been delineated for the Australian EEZ (HIMI). Extending the framework to the entire plateau, including HIMI and Banzare Bank, would provide a more complete connectivity picture across jurisdictions and allow us to assess whether the PF plays a similarly important role in regulating larval connectivity between EEZs.

## Author Contributions

**Fanny Ouzoulias:** writing – original draft, methodology, formal analysis, conceptualization, software, validation, visualization. **Alex Nalivaev:** methodology, software, formal analysis, validation, writing – original draft, visualization. **Clara Péron:** conceptualization, methodology, validation, supervision, funding acquisition, resources. **Amaël Dupaix:** methodology, writing – review and editing. **Éric Feunteun:** writing – review and editing, supervision. **Félix Massiot‐Granier:** conceptualization, methodology, supervision, validation, resources, writing – review and editing.

## Conflicts of Interest

The authors declare no conflicts of interest.

## Supporting information


**Table S1:** Parameters and fit of the model of female spawner abundance.
**Table S2:** Parameters and fit of the model of female spawner length.
**Table S3:** Parameter estimates of the Gaussian GLM relating log recruitment (logR) to standardized PF‐associated jet intensity ($z\left(I_{\mathrm{PF}}\right)$).
**Table S4:** Parameter estimates of the Gaussian GLM relating log recruitment (logR) to standardized SAF intensity ($z\left(I_{\mathrm{SAF}}\right)$), computed along the annual SAF streamline.
**Table S5:** Seasonal mixed‐layer‐depth context during the simulated larval‐drift period.
**Figure S1:** Positions of the Polar Front (PF) and Subantarctic Front (SAF) and definition of the sectors used to compute the front intensity indices. Grey lines show the annual PF and SAF streamlines (2000–2023); the dark red line is the 2000–2023 mean PF streamline and the blue line the 2000–2023 mean SAF streamline. The red box (67°–72° E, 48°–51° S) delineates the sector over which the PF‐associated jet intensity is computed along each annual PF streamline. The blue box (63°–73° E, 44°–47° S) delineates the sector over which SAF intensity is computed along each annual SAF streamline.
**Figure S2:** Inter‐weekly variability of larval retention for spawning events from week 22 to 30.
**Figure S3:** Sensitivity of larval dispersal outcomes to simulation duration.
**Figure S4:** Sensitivity of larval dispersal outcomes to depth range for suitable recruitment areas.
**Figure S5:** Larval density at the end of the simulation period, from 2000 to 2023.
**Figure S6:** Interannual variability in the PF‐associated streamline and larval dispersal patterns around the Kerguelen Plateau from 2000 to 2023. Blue lines show the annual PF streamline. Colours represent the time‐integrated density of egg‐weighted particles over the larval dispersal simulations.
**Figure S7:** Connectivity between spawning and recruitment areas over 2000–2023. Horizontal stacked bars show, for each spawning area, the mean percentage of eggs released in that area whose particles were retained after 18 weeks in each recruitment area. Percentages within the main bar segments indicate the corresponding spawning‐to‐recruitment connections. Values to the right of each bar give the mean total retention rate for that spawning area, with the minimum–maximum annual range in parentheses. Percentages below the spawning‐area names indicate the proportion of total egg production originating from each spawning area. NW: north‐western recruitment area; NE: north‐eastern recruitment area.
**Figure S8:** Pearson correlations among the annual metrics (2000–2023, n=24). Values in bold have p<0.05.

## Data Availability

The data and code that support the findings of this study are openly available in Seanoe at https://doi.org/10.17882/119264 and in a GitHub repository at https://github.com/fannyouzoulias/GCB‐dispersal‐toothfish. Surface geostrophic velocities used to force the Lagrangian simulations are openly available from the CNES‐CLS DUACS multi‐satellite altimetry product, distributed by the Copernicus Marine Service, at https://doi.org/10.48670/moi‐00148. Bathymetry and seabed slope were derived from the GEBCO Grid, openly available from the GEBCO Bathymetric Compilation Group 2024, British Oceanographic Data Centre, National Oceanography Centre, NERC, United Kingdom (https://www.gebco.net/). The mean positions of the Antarctic Circumpolar Current fronts are openly available on SEANOE at https://doi.org/10.17882/59800. The station‐based Southern Annular Mode index defined by Marshall (2003) is openly available from the British Antarctic Survey at https://legacy.bas.ac.uk/met/gjma/sam.html. Larval trajectories were computed with the LAMTA software (LAgrangian Manifolds Tracking Algorithm; Rousselet et al. 2025).
