# Supplementary material for: Climate‐Driven Oceanographic Changes Shape Larval Dispersal Success in the Southern Ocean
Source: Glob Chang Biol. 2026 Nov 5;32(11):e71168. doi: 10.1111/gcb.71168 (PMC13735991; doi:10.1111/gcb.71168)
Supplement: Supplementary file 1 [file GCB-32-e71168-s001.pdf]

Supporting Information

Climate-driven oceanographic changes shape larval dispersal success in the Southern Ocean

Fanny Ouzoulias, Alex Nalivaev, Clara Péron, Amaël Dupaix, Éric Feunteun, Félix Massiot-Granier

Appendix S1. Supplementary Methods

S1.1 Spawner abundance model

Spawner abundance was modeled using a Tweedie-distributed GLM with a log link:

$$N_{\text{spawners},i} \sim \text{Tweedie}(\mu_i, \phi, p) \tag{S1}$$

$$\log(\mu_i) = \alpha_0 + f_1(\text{Depth}_i) + \alpha_1 \text{Slope}_i + \alpha_2 \log(\text{Effort}_i) + u(s_i) \tag{S2}$$

where $u(s_i)$ is a spatial Gaussian Markov Random Field with Matérn covariance. f_1 denotes a smooth, non-linear effect of depth, modeled using a penalized thin plate regression spline. A constrained Delaunay triangulation mesh with a minimum edge length of 10 km and coastline barriers was used. Model diagnostics and parameter estimates are provided in Table S1.

Interactions among covariates were tested individually. The deviance explained for each model was considered as an indication of the quality of fit, as well as scrutiny of the distribution of model residuals. This model was then used to predict the distribution of female spawner abundance over the Kerguelen Plateau across the observed depth range (508-2120 m). The generalization dataset included latitude, longitude and depth from the 'GEBCO 2024' bathymetric grid, at a horizontal resolution of 0.0083°x 0.0083°, `https://www.gebco.net/data-products/historical-data-sets#gebco_2024`). Slope was calculated using the GEBCO 2024 bathymetric grid using the function *terrain* in R software. The fishing effort was fixed at 7251 hooks for predictions (mean of the dataset). The spatial resolution of the prediction grid was 10 x 10 km.

S1.2 Spawner total length model

Spatial variation in female total length was modeled using a Gaussian spatial model:

$$\log(\mathrm{TL}_i) \sim \mathcal{N}(\eta_i, \sigma^2) \tag{S3}$$

$$\eta_i = \beta_0 + g_1(\mathrm{Depth}_i) + \beta_2 \mathrm{Slope}_i + u(s_i) \tag{S4}$$

using the same spatial mesh and barrier structure as the abundance model. g_1 denotes a smooth, non-linear effect of depth, modeled using a penalized thin plate regression spline. Parameter estimates are reported in Table S2.

Interactions among covariates were tested individually. The deviance explained for each model was considered as an indication of the quality of fit, as well as scrutiny of the distribution of model residuals. Model predictions were generated with the same initial grid as for the abundance model (Appendix S1.1).

S1.3 Fecundity–length relationship

For each mature female, egg volume (V_{egg}) was approximated by assuming spherical eggs:

$$V_{\mathrm{egg}} = \frac{4}{3}\pi \left(\frac{D}{2}\right)^3,$$

where D is the measured egg diameter. Individual fecundity (N_{eggs}) was then derived from the measured gonad volume (V_{gonad}) as:

$$N_{\mathrm{eggs}} = \frac{V_{\mathrm{gonad}}}{V_{\mathrm{egg}}}.$$

We fitted an exponential fecundity–length relationship to stage-4 females using non-linear least squares:

$$N_{\mathrm{eggs},i} = a \exp(b \cdot \mathrm{TL}_i),$$

where TL_i is total length (cm), and a and b are fitted parameters. Uncertainty around the fitted relationship (for visualization of the curve) was quantified using a first-order Taylor (delta-method) approximation based on the variance–covariance matrix of (a, b).

For spatial predictions, fecundity uncertainty was propagated by applying the fitted relationship to posterior simulations of predicted total length from the spatial length model. Specifically, for each grid cell i, we computed

$$F_{i,s} = a \exp(b \cdot \mathrm{TL}_{i,s}), \qquad s = 1, \ldots, 1000,$$

where $\mathrm{TL}_{i,s}$ denotes the s-th posterior simulation of predicted total length at cell i. The resulting distribution $\{F_{i,s}\}$ was summarized by its mean and standard deviation, yielding spatial layers of mean fecundity and associated uncertainty.

S1.4 Mapping egg production

Spatial egg production was estimated on a 10 × 10 km grid by combining (i) predicted female spawner abundance from the spatiotemporal abundance model and (ii) fecundity predicted from the spatial length model and the length–fecundity relationship (Appendix S1.3). To propagate uncertainty from both components, we generated 1000 posterior predictive simulations of female abundance, $N_{i,s}$, and used the 1000 fecundity simulations $F_{i,s}$ described above.

For each grid cell i and simulation s, total egg production was computed as:

$$E_{i,s} = N_{i,s} \times F_{i,s}, \qquad s = 1, \ldots, 1000.$$

This yielded 1000 realizations of egg production per grid cell. For each cell, we reported the mean and standard deviation of $\{E_{i,s}\}$ as spatially explicit estimates of egg production and associated uncertainty during the spawning period.

S1.5 Construction of the annual Polar Front and SAF streamlines and associated jet intensity

S1.5.1 Reference Polar Front streamline

Park et al. (2019) represented the major ACC fronts as climatological surface geostrophic streamlines derived from the CNES–CLS18 mean dynamic topography (MDT). In this product, the PF corresponded to the −0.58 m MDT contour and, around Kerguelen, followed a characteristic pathway south of the islands and along the southern and eastern 500–1000 m escarpment. Because the original CNES–CLS18 MDT product used by Park et al. (2019) is no longer the current product, we transferred this reference pathway to the more recent CNES–CLS22 MDT product (doi: 10.48670/moi-00150), rather than applying the original −0.58 m contour value directly. CNES–CLS22 MDT values were sampled along the published PF pathway (Park and Durand, 2019), and their median was used as the CNES–CLS22 reference PF value. We verified that the corresponding climatological contour reproduced the PF pathway described by Park et al. (2019) around Kerguelen.

S1.5.2 Annual PF streamline construction

For each year from 2000 to 2023, daily DUACS absolute dynamic topography (ADT) fields were extracted over the larval-advection period, from the first surface release in

early June to the end of the last 18-week trajectory in mid-December. To place ADT and MDT on a consistent regional reference level, we calculated the difference between the daily mean ADT and the mean CNES–CLS22 MDT over 55–85°E and 46–56°S and subtracted this difference uniformly from each daily ADT field. The adjusted daily fields were then averaged over the larval-advection period. We extracted the ADT contour corresponding to the CNES–CLS22 reference PF value defined above. To preserve the Kerguelen pathway described by Park et al. (2019), the selected streamline was required to pass south of Kerguelen and to cross the 500–1000 m escarpment at 69°E. When the reference contour did not satisfy this condition, neighbouring ADT contours were tested in 0.001 m increments, up to ±0.05 m, and the closest contour satisfying the pathway criterion was retained.

S1.5.3 PF-associated jet intensity

We quantified PF-associated jet intensity as the mean surface geostrophic current speed along the annual PF streamline within 67–72°E and 48–51°S. At each point along the streamline, current speed was calculated from the time-mean zonal and meridional geostrophic velocity components over the larval-advection period:

$$U = \sqrt{\overline{u}^2 + \overline{v}^2}, \tag{S5}$$

where $\overline{u}$ and $\overline{v}$ are the mean zonal and meridional surface geostrophic velocities over the larval-advection period. PF-associated jet intensity was then calculated as the mean of U along the annual PF streamline within the specified sector.

S1.5.4 Annual SAF streamline and intensity

The Subantarctic Front (SAF) was represented in the same way as the PF. Its climatological pathway was taken from Park and Durand (2019), in which it corresponds to the −0.10 m contour of the CNES–CLS18 MDT (Park et al., 2019). As for the PF, the published pathway was transferred to CNES–CLS22: the median of the CNES–CLS22 MDT along the published SAF within the regional domain (−0.079 m) defined the reference SAF value. For each year, the annual SAF streamline was the contour at this value of the mean ADT field of the larval-advection period, adjusted, averaged and smoothed as described above for the PF. No pathway criterion was applied, since the SAF flows in deep water about 350 km north of Kerguelen Island, away from the escarpment that constrains the PF.

SAF intensity was calculated as the mean of U along the annual SAF streamline within 63–73°E and 44–47°S.

S1.6 Metrics describing the larval dispersal pathways

To complement trajectory-density maps, we derived annual metrics quantifying transport behaviour and pathway occupancy.

(A) Corridor-crossing indices. To quantify the relative use of contrasted advection pathways, we defined three geographical corridors and computed, for each year, the number of particles that entered each corridor at least once during the simulation window. A particle was considered to have crossed a corridor if any of its positions fell within the corridor's bounding box:

- **NW corridor:** 68.5–69.5°E and 49.25–47°S.
- **NE corridor:** 69.5–71°E and 49.25–47°S.
- **Southern corridor:** 69–71°E and 51–49.5°S.

The NW and NE corridors correspond to the northern shelf pathway and the southern corridor to the PF-associated southern pathway. These indices are used in the main text (Results, section 'Larval dispersal pathways') to relate the number of particles following each pathway to frontal intensity and to the trapping index.

(B) Trapping in the western recirculation cell. We defined a western recirculation neighborhood (63–67°E and 50–48°S) and quantified the fraction of particles that became trapped in this region. A particle was classified as trapped if (i) it entered the neighborhood at least once and (ii) all subsequent positions remained inside the neighborhood until the end of the simulation. The annual trapped fraction was computed as the percentage of trapped particles among all released particles (weeks 24–28).

(C) Southern Annular Mode (SAM) index. As a large-scale descriptor of the extratropical atmospheric circulation, we used the observation-based, station-derived SAM index defined by Marshall (2003). This index is a continuously updated time series maintained by the British Antarctic Survey; monthly values (1957–2023) were obtained from `https://legacy.bas.ac.uk/met/gjma/sam.html`. From the monthly series we derived an annual mean index, seasonal means (DJF, MAM, JJA, SON, with December assigned to the following year's DJF), and a June–December mean spanning the spawning and dispersal period. These indices were used to relate frontal intensity and larval retention to large-scale atmospheric forcing.

Over the 24-year series, the annual SAM index was positively but only marginally correlated with PF-associated jet intensity ($r = 0.38$, $p = 0.068$, $n = 24$), and was uncorrelated with Subantarctic Front intensity ($r = 0.09$, $p = 0.68$). The SAM–PF relationship depended on the season: it was significant in austral winter and spring (JJA: $r = 0.46$, $p = 0.024$; SON: $r = 0.46$, $p = 0.022$) but weak in summer and autumn (DJF and MAM:

$|r| \leq 0.26$, $p > 0.22$). When all seasons were pooled in a single model relating standardized PF-associated jet intensity to the seasonal SAM index and season, the SAM effect was positive but not significant ($p = 0.062$). The annual SAM index was not directly correlated with larval retention (log number of retained larvae; $r = -0.29$, $p = 0.18$), whereas PF-associated jet intensity was ($r = -0.49$, $p = 0.015$).

(D) Trajectory re-entry analysis. Because retention is assessed from each particle's final position, particles can leave a recruitment area and later be transported back into one. Across all particles released during weeks 24–28 over 2000–2023 ($n = 316{,}920$), 11.0% entered a recruitment area, subsequently left all recruitment areas, and later re-entered (unweighted count). The resulting excursions were generally short and local: across 50,082 individual excursions, the median duration was 4 days and the median distance traveled outside recruitment habitat was 15 km; 74% covered less than 50 km and 13% exceeded 100 km before re-entry.

We also quantified how much this process contributed to the sensitivity to pelagic larval duration, by separating, at each duration, the particles inside recruitment habitat into those that had never left it and those that had left and returned. Using the same egg-production weighting as in the main analysis, retention increased from 8.6% after 10 weeks to 19.3% after 18 weeks, a difference of 10.7 percentage points. Of this net increase, 3.5 percentage points (33%) were associated with particles that had left recruitment habitat and subsequently returned. Thus, re-entry contributes to the higher retention obtained with longer simulations, but accounts for only about one third of the increase between 10 and 18 weeks.

Correlation across metrics. Pairwise associations among annual metrics were explored using Pearson correlations. They are shown in Figure S8. Each correlation was calculated across the 24 simulation years (2000–2023). The matrix is intended as a descriptive overview of covariation among metrics.

S1.7 Larval retention metrics

(1) Annual larval retention rate. The annual larval retention rate was computed as:

$$\text{Retention}(Y) = 100 \times \frac{\text{particles released in year } Y \text{ that reach any suitable recruitment area}}{\text{all particles released in year } Y}.$$

We then derived two complementary percentage metrics to describe the spatial structure of larval retention, based on weighted particles.

(2) Spatial distribution of retention by recruitment zone.

$$\text{Percent}_{\text{rec}}(Y, R) = 100 \times \frac{\text{particles released in year } Y \text{ that end in recruitment zone } R}{\text{all particles released in year } Y \text{ (all spawning zones)}}$$

This measures, for each year Y, how the retained particles are distributed among recruitment zones R.

(3) Contribution of each spawning area to the overall retention.

$$\text{Percent}_{\text{spawn}}(Y, S) = 100 \times \frac{\text{particles from spawning zone } S \text{ that reach any recruitment zone in year } Y}{\text{all particles released in year } Y \text{ (all spawning zones)}}.$$

This measures, for each year Y, how much each spawning zone S contributes to the total number of particles that reach recruitment areas.

(4) Connectivity between spawning and recruitment areas.

$$\text{Percent}_{\text{conn}}(S, R) = 100 \times \frac{\text{particles from spawning zone } S \text{ that end in recruitment zone } R}{\text{all particles released in spawning zone } S}.$$

This measures the retention rate of each spawning zone S and how it is distributed among recruitment zones R, independently of the share of total egg production released in S.

Supplementary Tables

Table S1: Parameters and fit of the model of female spawner abundance.

Parameter	Coefficient Estimate	Standard Error
(Intercept)	-7.20	2.73
Slope	0.19	0.07
log(Effort)	0.93	0.31
s(Depth)	0.08	0.60

Percentage of deviance explained: 54%.

Table S2: Parameters and fit of the model of female spawner length.

Parameter	Coefficient Estimate	Standard Error
(Intercept)	4.69	0.01
Slope	0.01	0.01
s(Depth)	0.04	0.03

Percentage of deviance explained: 26%.

Table S3: Parameter estimates of the Gaussian GLM relating log recruitment ($\log R$) to standardized PF-associated jet intensity ($z(I_{PF})$).

Parameter	Coefficient Estimate	Standard Error	p-value
(Intercept)	23.04	0.06	$< 2 \times 10^{-16}$
$z(I_{PF})$	-0.16	0.06	0.015

Table S4: Parameter estimates of the Gaussian GLM relating log recruitment ($\log R$) to standardized SAF intensity ($z(I_{SAF})$), computed along the annual SAF streamline.

Parameter	Coefficient Estimate	Standard Error	p-value
(Intercept)	23.04	0.07	$< 2 \times 10^{-16}$
$z(I_{SAF})$	0.08	0.07	0.272

Table S5: Seasonal mixed-layer-depth context during the simulated larval-drift period.

**Month**	**Simulation window**	**% of particles drifting during that month**	**MLD state**	**MLD around Kerguelen**	**Implication for our study**	**Sources**
June	7–30 June	3.8	Winter deepening	The mixed layer is deepening toward its late-winter maximum. Monthly values around Kerguelen are less well constrained for June than for late winter.	Only 3.8% of the simulated drift occurs in June.	(Park et al., 1998, Doriot et al., 2025, DuVivier et al., 2018)
July	Full month	15.6	Continued deepening	The mixed layer continues to deepen toward late-winter values. Around Kerguelen, winter MLD approaches the 150–200 m range reached later in the season.	A substantial part of the upper water column is increasingly mixed, although larvae near 250 m may still lie below the mixed layer.	(Park et al., 1998, Doriot et al., 2025, DuVivier et al., 2018)
August	Full month	24.1	Late-winter maximum	At KERFIX (50°40′S, 68°25′E), monthly mean MLD reaches 185 m in August.	August represents 24.1% of the simulated drift. Surface currents are therefore likely to represent transport over a relatively large fraction of the upper water column during an important part of the simulations.	(Park et al., 1998, Doriot et al., 2025)
September	Full month	23.6	Maximum / near maximum	Late-winter MLD around Kerguelen is typically of order 150–200 m.	September represents 23.6% of the simulated drift. Larvae in the upper ∼150–200 m may remain within the mixed layer, whereas individuals near 250 m may be below it depending on location.	(Park et al., 1998, Doriot et al., 2025, DuVivier et al., 2018, Caneill et al., 2024)
October	Full month	21.7	Deep, then shoaling	The mixed layer remains relatively deep early in October and then shoals as spring restratification develops.	October represents 21.7% of the simulated drift. The surface-flow approximation becomes progressively less representative for deeper larvae as the mixed layer shoals.	(Park et al., 1998, Doriot et al., 2025)

Continued on next page

Table S5 – continued from previous page

Month	Simulation window	% of particles drifting during that month	MLD state	MLD around Kerguelen	Implication for our study	Sources
November	Full month	10.2	Spring restratification	The mixed layer is shallower and more spatially variable than during the late-winter maximum.	For the 10.2% of simulated drift occurring in November, deeper larvae are more likely to occur below the mixed layer and to experience currents differing from surface flow.	(Doriot et al., 2025, Bowie et al., 2015)
December	1–12 December	0.9	Shallow spring / early summer	The mixed layer approaches its shallow summer state; at KERFIX, the January monthly mean MLD is about 60 m, providing an indication of the subsequent summer minimum.	Only 0.9% of the simulated drift occurs in December. Larvae at depth would generally be below the shallow mixed layer during this period.	(Park et al., 1998, Doriot et al., 2025)

Interpretation. MLD around Kerguelen varies strongly through the June–December simulation period and cannot be represented by a single value. It reaches approximately 150–200 m in late winter and then shoals during spring. Larvae near 250 m may occur below the mixed layer, especially during spring. Importantly, 69.4% of the simulated larval-drift exposure occurs from August to October, when the mixed layer is deepest or only beginning to shoal, whereas June and December together account for only 4.7%.

Supplementary Figures

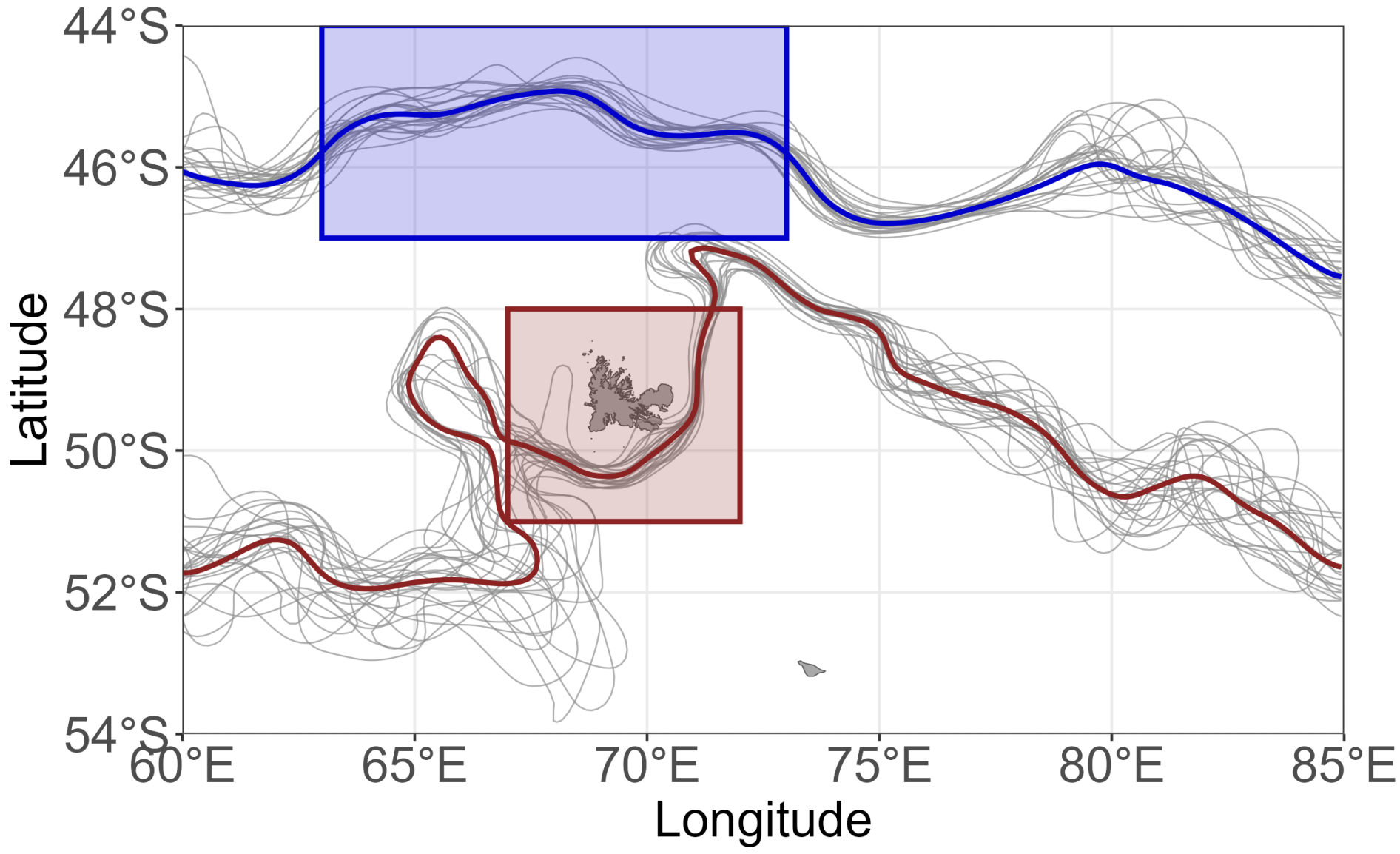

Figure S1: Positions of the Polar Front (PF) and Subantarctic Front (SAF) and definition of the sectors used to compute the front intensity indices. Grey lines show the annual PF and SAF streamlines (2000–2023); the dark red line is the 2000–2023 mean PF streamline and the blue line the 2000–2023 mean SAF streamline. The red box (67–72°E, 48–51°S) delineates the sector over which the PF-associated jet intensity is computed along each annual PF streamline. The blue box (63–73°E, 44–47°S) delineates the sector over which SAF intensity is computed along each annual SAF streamline.

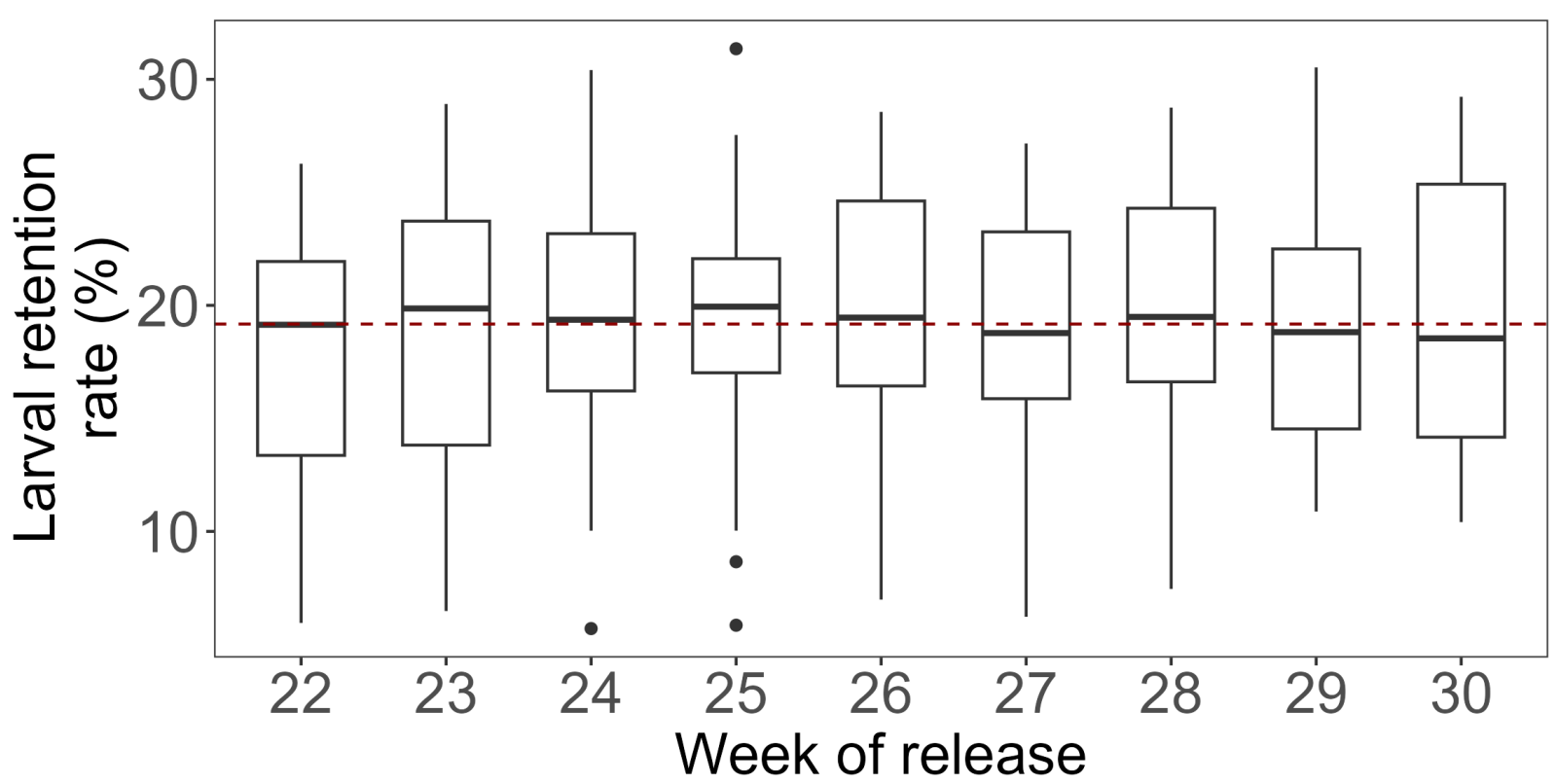

Figure S2: Inter-weekly variability of larval retention for spawning events from week 22 to 30.

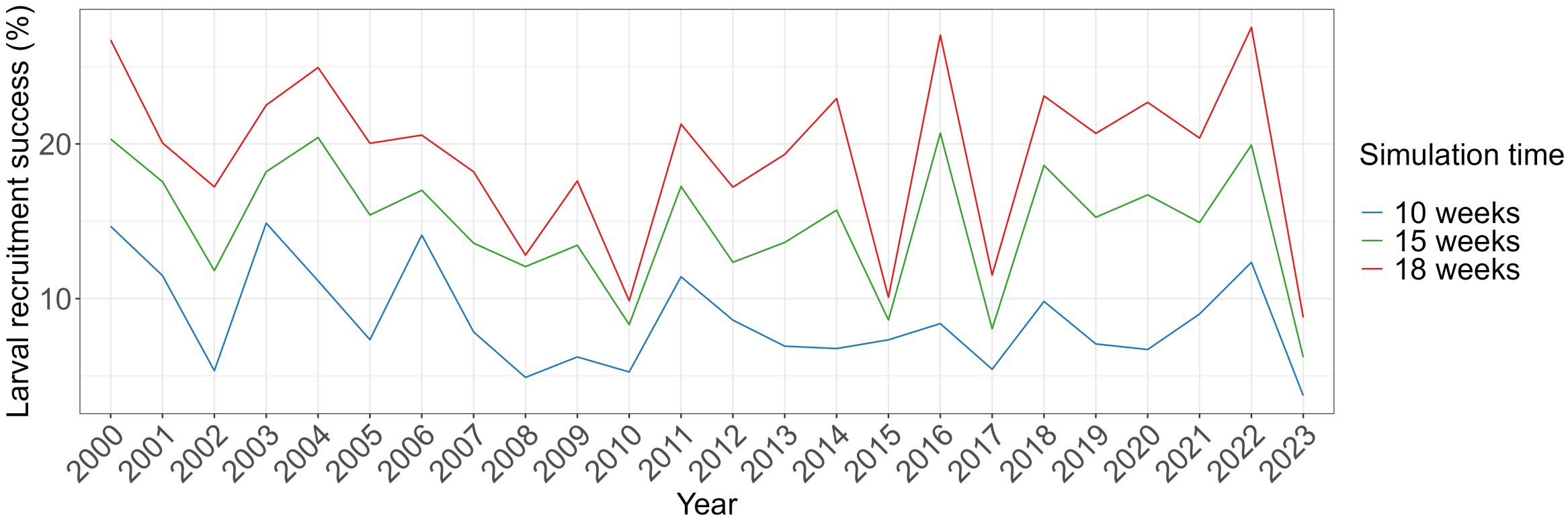

Figure S3: Sensitivity of larval dispersal outcomes to simulation duration.

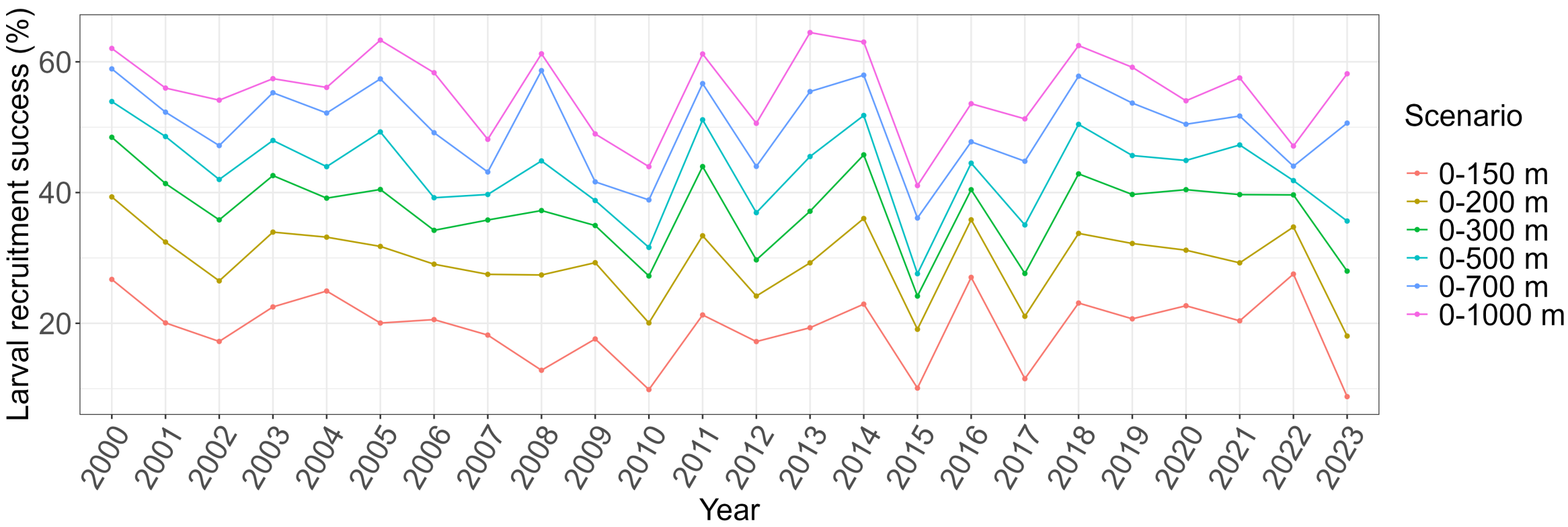

Figure S4: Sensitivity of larval dispersal outcomes to depth range for suitable recruitment areas.

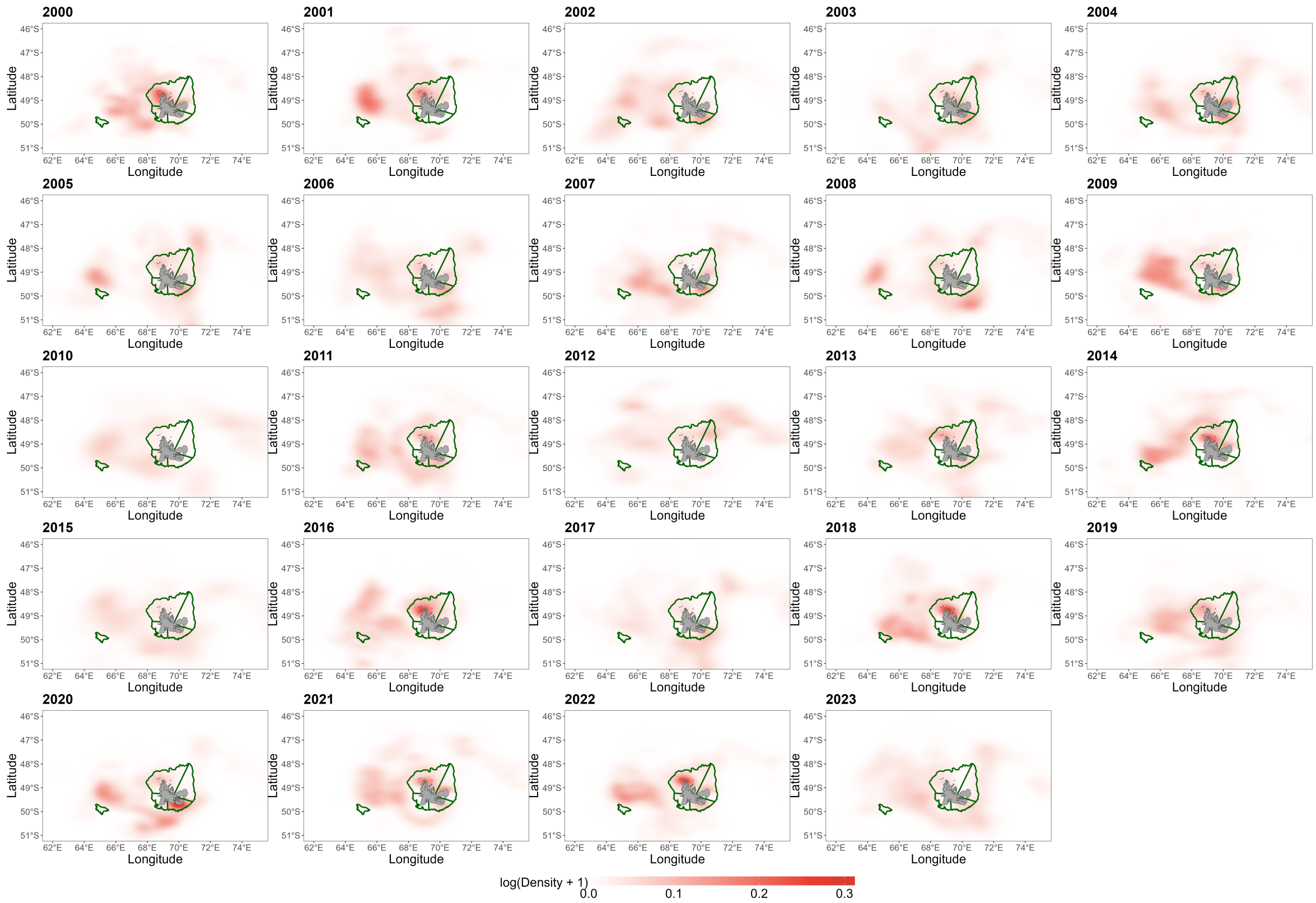

Figure S5: Larval density at the end of the simulation period, from 2000 to 2023.

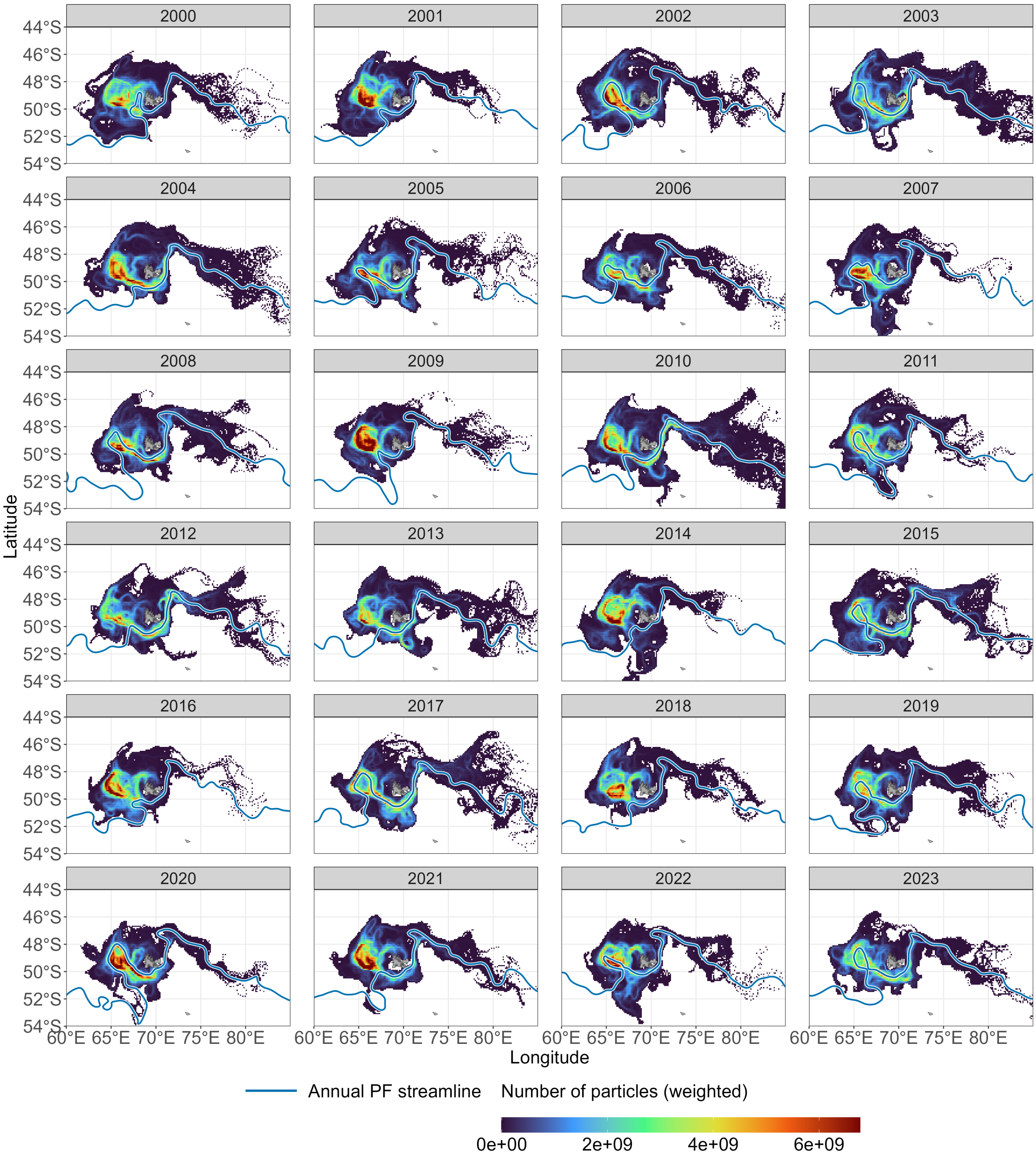

Figure S6: Interannual variability in the PF-associated streamline and larval dispersal patterns around the Kerguelen Plateau from 2000 to 2023. Blue lines show the annual PF streamline. Colours represent the time-integrated density of egg-weighted particles over the larval dispersal simulations.

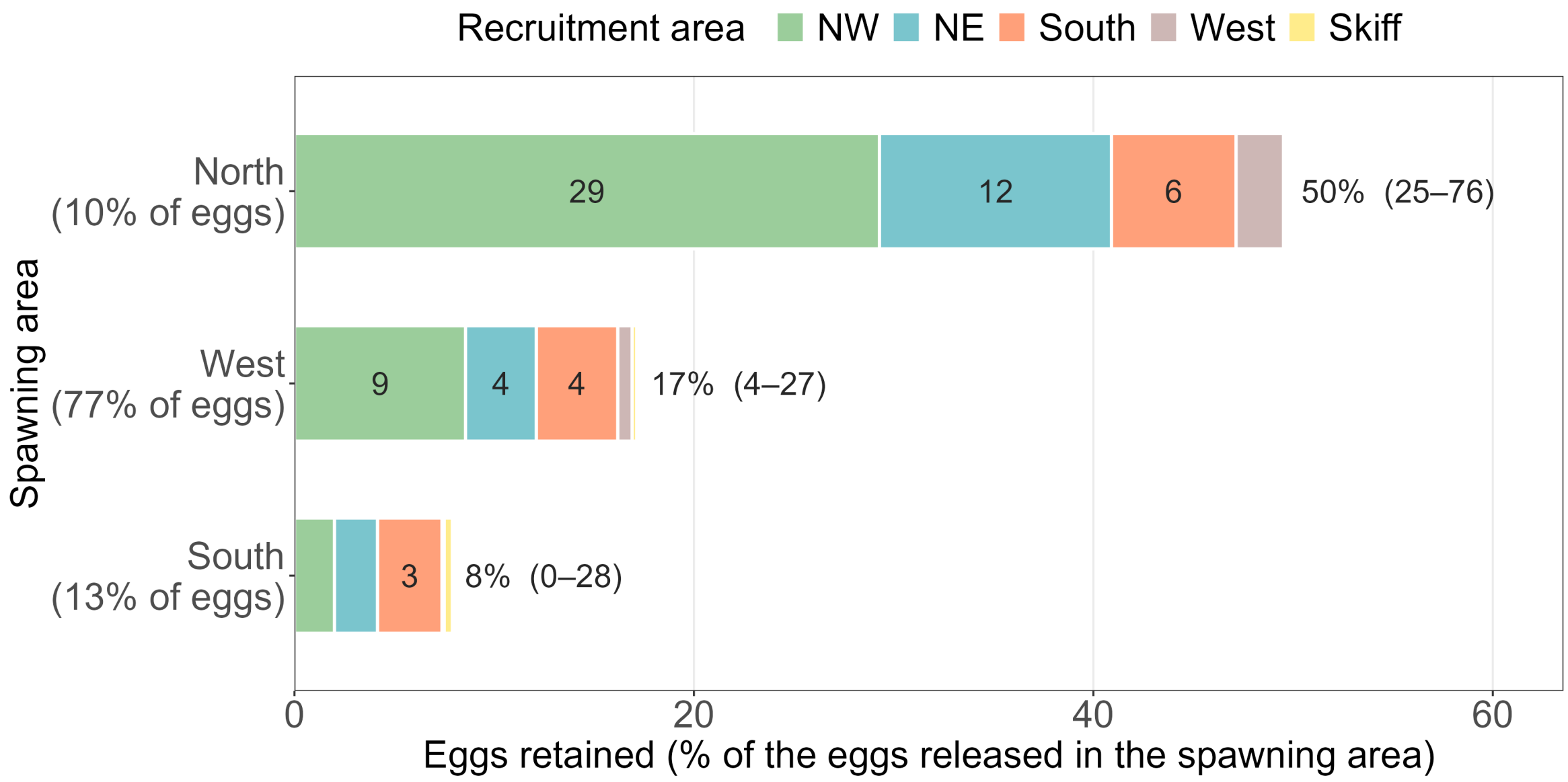

Figure S7: Connectivity between spawning and recruitment areas over 2000–2023. Horizontal stacked bars show, for each spawning area, the mean percentage of eggs released in that area whose particles were retained after 18 weeks in each recruitment area. Percentages within the main bar segments indicate the corresponding spawning-to-recruitment connections. Values to the right of each bar give the mean total retention rate for that spawning area, with the minimum–maximum annual range in parentheses. Percentages below the spawning-area names indicate the proportion of total egg production originating from each spawning area. NW: north-western recruitment area; NE: north-eastern recruitment area.

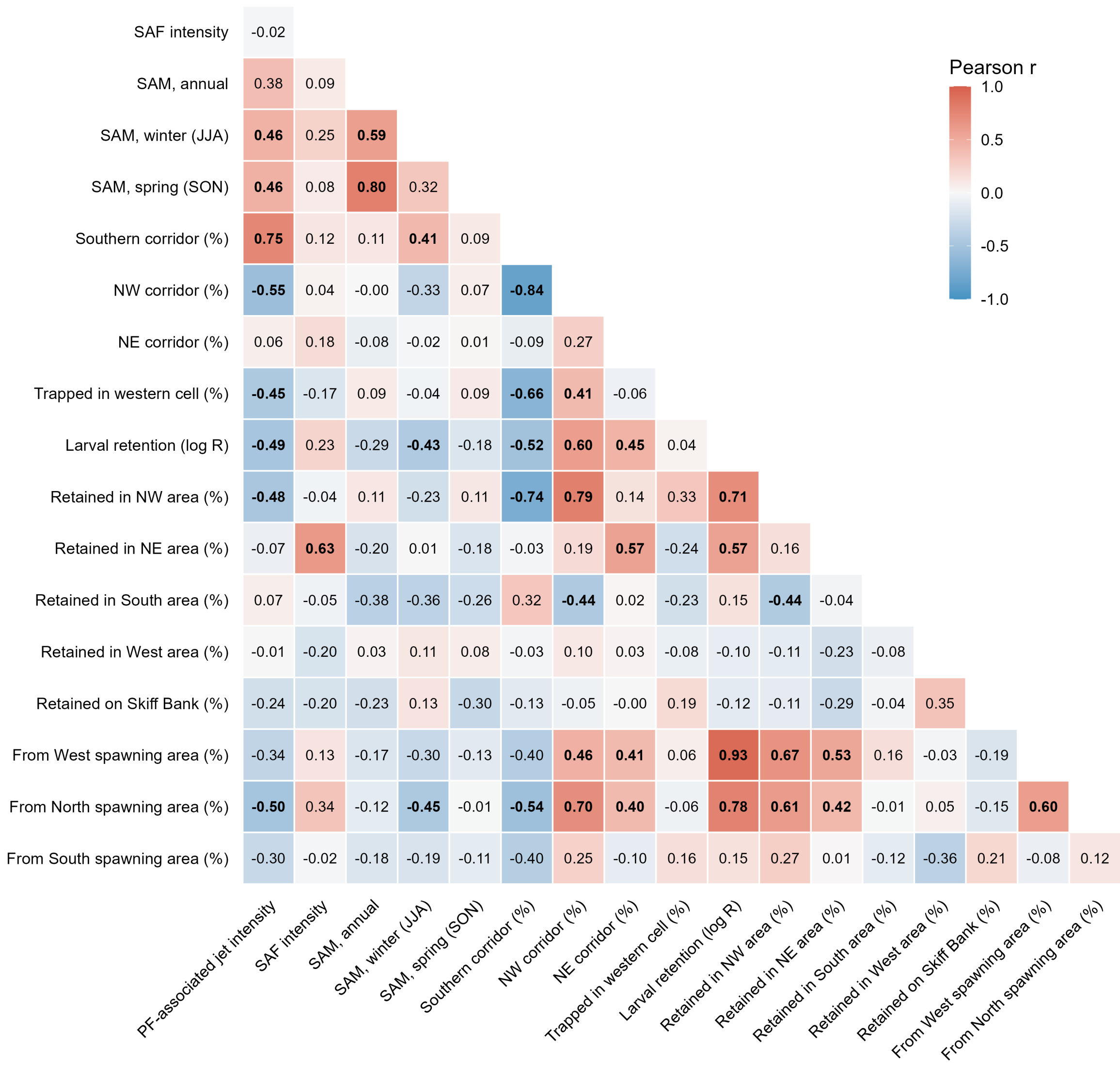

Figure S8: Pearson correlations among the annual metrics (2000–2023, $n = 24$). Values in bold have $p < 0.05$.

References

Bowie, A. R., van der Merwe, P., Quéroué, F., Trull, T., Fourquez, M., Planchon, F., Sarthou, G., Chever, F., Townsend, A. T., Obernosterer, I., Sallée, J.-B., and Blain, S. (2015). Iron budgets for three distinct biogeochemical sites around the Kerguelen Archipelago (Southern Ocean) during the natural fertilisation study, KEOPS-2. *Biogeosciences*, 12(14):4421–4445.

Caneill, R., Roquet, F., and Nycander, J. (2024). The Southern Ocean deep mixing band emerges from a competition between winter buoyancy loss and upper stratification strength. *Ocean Science*, 20(2):601–619.

Doriot, V., Roquet, F., Nerini, D., Caneill, R., Picard, B., and Guinet, C. (2025). Distribution and recent trends of the Mixed Layer Depth around the Kerguelen Plateau. *ESS Open Archive*, (0114).

DuVivier, A. K., Large, W. G., and Small, R. J. (2018). Argo Observations of the Deep Mixing Band in the Southern Ocean: A Salinity Modeling Challenge. *Journal of Geophysical Research: Oceans*, 123(10):7599–7617.

Marshall, G. J. (2003). Trends in the Southern Annular Mode from Observations and Reanalyses. *Journal of Climate*, 16(24):4134–4143.

Park, Y. H., Charriaud, E., Pino, D. R., and Jeandel, C. (1998). Seasonal and interannual variability of the mixed layer properties and steric height at station KERFIX, southwest of Kerguelen. *Journal of Marine Systems*, 17(1):571–586.

Park, Y.-H. and Durand, I. (2019). Altimetry-drived Antarctic Circumpolar Current fronts.

Park, Y.-H., Park, T., Kim, T.-W., Lee, S.-H., Hong, C.-S., Lee, J.-H., Rio, M.-H., Pujol, M.-I., Ballarotta, M., Durand, I., and Provost, C. (2019). Observations of the Antarctic Circumpolar Current Over the Udintsev Fracture Zone, the Narrowest Choke Point in the Southern Ocean. *Journal of Geophysical Research: Oceans*, 124(7):4511–4528.
